# Biomembrane-coated Nanoparticles Targeting circHIF1α Suppress Ovarian Cancer Metastasis and Cisplatin Resistance by Mediating System Xc⁻ Inactivation via SLC7A11/SLC3A2 to Induce Ferroptosis in Cancer Stem Cells

**DOI:** 10.7150/ijbs.130412

**Published:** 2026-05-18

**Authors:** Yinyi Chang, Jingjing Wang, Liying Ma, Yi Liu, Yumeng Zhu, Yu Yi, Dongdong Zhang, Zitong Zhao, Li Sun, Yongmei Song

**Affiliations:** 1 State Key Laboratory of Molecular Oncology, National Cancer Center/National Clinical Research Center for Cancer/Cancer Hospital, Chinese Academy of Medical Sciences and Peking Union Medical College, Beijing, 100021, China.; 2 National Cancer Center/National Clinical Research Center for Cancer/Cancer Hospital & Shenzhen Hospital, Chinese Academy of Medical Sciences and Peking Union Medical College, Shenzhen, 518116, China.; 3 State Key Laboratory of Epigenetic Regulation and Intervention, Institute of Biophysics, Chinese Academy of Sciences, Beijing, 100101, China.; 4 CAS Center for Excellence in Nanoscience, CAS Key Laboratory for Biomedical Effects of Nanomaterials and Nanosafety, National Center for Nanoscience and Technology (NCNST), Beijing, 100190, China.; 5 College of Arts & Sciences, Boston University, Boston, MA, 02215, USA.

**Keywords:** ovarian cancer, chemotherapy resistance, cancer stem cells, ferroptosis, exosomes, nanoparticles

## Abstract

The hypoxic tumor microenvironment (TME) drives malignant progression by increasing the activity of cancer stem cells (CSCs), whose iron-dependent metabolism not only maintains their stemness but also promotes drug resistance and ferroptosis resistance by activating key signaling pathways. This study revealed that hypoxia induces circHIF1α expression in CSCs and that its expression is significantly associated with chemotherapy resistance in patients. Functional experiments confirmed that circHIF1α enhances CSC stemness by regulating iron metabolism and driving cancer progression. The mechanism involves the dual regulation of ferroptosis: binding SLC3A2 to block lysosomal degradation and sponging miR-375 to abrogate its inhibition of SLC7A11, synergistically activating system Xc⁻-mediated glutathione synthesis for ferroptosis resistance. Additionally, circHIF1α can be transferred between cells in the TME via exosomes, promoting iron metabolic reprogramming and the spread of drug resistance. Based on this mechanism, we developed a cell membrane-coated siRNA nanodelivery system targeting circHIF1α and confirmed that the combination of this system with cisplatin exerted a synergistic antitumor effect. This study demonstrated that circHIF1α maintains ferroptosis resistance and a “high-iron metabolic” state in CSCs by preserving the antioxidant barrier, providing novel insights into the mechanism of circular RNAs and potential targets/strategies for overcoming chemotherapy resistance in ovarian cancer.

## Introduction

Ovarian cancer is one of the most common malignancies of the female reproductive system and has the highest mortality rate among gynecological cancers 1,2. Epithelial ovarian cancer (EOC) accounts for approximately 90% of ovarian cancer cases 3,4. Due to the lack of specific symptoms, the early diagnosis of EOC remains a major challenge, and the majority of patients are diagnosed at an advanced stage 5. The standard treatment for advanced EOC comprises maximal cytoreductive surgery combined with platinum- and taxane-based chemotherapies 6. Although surgical techniques, chemotherapy regimens, and maintenance therapies have improved in recent years (e.g., PARP inhibitors have become the standard maintenance treatment for ovarian cancer 7), the recurrence rate among patients with advanced-stage tumors remains exceedingly high (>70%) 8. More critically, as patients undergo successive lines of therapy, platinum resistance frequently develops 9, leading to progressively shorter remission durations. The five-year survival rate of patients with ovarian cancer is less than 50% 10. Thus, drug resistance continues to be a major obstacle in the treatment of EOC. Identifying the key molecular drivers and underlying mechanisms of drug resistance is of paramount importance 11.

Circular RNAs (circRNAs) are a class of covalently closed circular noncoding RNA molecules characterized by high stability and tissue-specific expression patterns 12,13. They can be significantly upregulated in response to characteristic pathological stresses within the tumor microenvironment (TME), such as hypoxia, nutrient deprivation, oxidative stress, and metabolic dysregulation. circRNAs participate in multiple pathways that drive tumorigenesis and cancer progression 14,15. Notably, tumor cells secrete circRNAs via exosomes, which deliver these molecules to various cell types in the microenvironment, including immune, stromal, and vascular endothelial cells 16. This exosome-mediated transfer of circRNAs not only facilitates intricate intercellular communication 17 but also plays important roles in key pathological processes such as tumor immune evasion 18, angiogenesis 19, and premetastatic niche formation 20. Liquid biopsy-based analysis of exosomes has emerged as a promising noninvasive strategy for cancer diagnosis, prognosis, and therapeutic monitoring 21. Exosomal circRNAs have been increasingly recognized as potential biomarkers for early cancer detection and a dynamic evaluation of the treatment response. Therefore, a deeper understanding of circRNA mechanisms will help elucidate the intrinsic processes underlying drug resistance in ovarian cancer. Given that cancer stem cells (CSCs) are pivotal to resistance to chemotherapy 22, the regulation of CSC stemness by circRNAs has emerged as a critical area of investigation.

CSCs are key factors in chemoresistance. Hypoxia enhances the self-renewal capacity of CSCs by activating hypoxia-inducible factor 1 subunit alpha (HIF1α) signaling 23,24. CSCs are central to platinum resistance and disease recurrence in EOC 25. Dysregulated iron metabolism is a notable characteristic of CSCs 26. Through the interaction of CD44 with the transferrin receptor (TFRC), CSCs promote iron uptake, while downregulating the expression of the iron exporter solute carrier family 40 member 1 (SLC40A1) to reduce iron efflux, establishing a distinct “high-iron accumulation” state 27. Despite the elevated iron levels, CSCs exhibit resistance to ferroptosis, suggesting that metabolic reprogramming maintains iron homeostasis. These findings offer a novel strategy for targeting iron metabolism in CSCs to increase chemosensitivity. In pancreatic ductal adenocarcinoma (PDAC), circRREB1 interacts with PGK1 to increase glycolytic activity and with YBX1 to maintain stemness properties 28. However, the roles of circRNAs in the regulation of iron metabolism and stemness in ovarian cancer remain unclear.

In this study, we elucidated a novel mechanism by which circHIF1α, a circRNA whose expression is induced by HIF1α under hypoxic conditions, promotes platinum resistance in EOC by modulating iron metabolism pathways linked to stemness traits. Mechanistically, circHIF1α activates system Xc⁻ to confer ferroptosis resistance and remodel iron metabolism, thereby enhancing CSC properties and driving the transition from a platinum-sensitive phenotype to a platinum-resistant phenotype, which facilitates metastasis and chemoresistance. Notably, circHIF1α can be transferred within the TME via exosomes, amplifying the dissemination of drug resistance. We developed biomimetic nanoparticles coated with homologous cancer cell membranes (CCMs) to target this pathway. These nanoparticles not only enable the efficient delivery of an siRNA targeting circHIF1α but also retain source cell-surface recognition molecules, achieving precise targeting and increased accumulation in tumors. Both *in vitro* and *in vivo* experiments showed the excellent targeting ability and potent antitumor efficacy of these nanoparticles. Our study elucidates the role of the circHIF1α-iron metabolism-stemness axis in driving platinum resistance in EOC and establishes a biomimetic nanotherapeutic strategy, providing important theoretical and practical foundations for understanding ovarian cancer resistance and advancing circRNA-targeted therapy.

## Materials and Methods

### Cell culture and transfection

A2780 and OVCAR3 cells were cultured in Roswell Park Memorial Institute-1640 (RPMI-1640) medium supplemented with 10% fetal bovine serum (FBS) and antibiotics. CAOV3 and SKOV3 cells were cultured in Dulbecco's modified Eagle's medium (DMEM) supplemented with 10% FBS and antibiotics. CAOV4 cells were cultured in DMEM supplemented with 20% FBS and antibiotics. All the cell lines were maintained and cultured in an incubator at 37 °C and 5% CO_2_. siRNAs targeting circHIF1α (CAGAACTTATCCATTTCTGTGT) and their negative controls were provided by JTSBIO Co., Ltd. (Wuhan, China). The miR-375 mimic or mi-NC was purchased from RiboBio (Guangzhou, China). Cells were transfected with siRNAs or microRNAs (miRNAs) using Lipofectamine 2000 (Invitrogen, 11668019). The pCDH-CiR-circHIF1α, pmirGLO-circHIF1α WT/MUT, and pmirGLO-SLC7A11 3'UTR WT/MUT plasmids were constructed by GeneRay, Inc. (Shanghai, China). The pGL4.2-HRE-luciferase plasmid was constructed by Mailgene (Beijing, China). The cells were transfected with plasmids using Neofect^TM^ DNA transfection reagent (NEOFECT, TF20121201).

### Western blot

Western blot was performed as previously described 29. The assays were performed using the following antibodies: anti-HIF1α (Proteintech, 20960-1-AP), anti-β-actin (Sigma, USA, A5316), anti-SLC40A1 (Proteintech, 26601-1-AP), anti-TFRC/CD71 (Proteintech, 66180-1-Ig), anti-FTH1 (Proteintech, 11682-1-AP), anti-SLC3A2/CD98 (Abcam, ab244356), anti-SLC7A11 (Proteintech, 26864-1-AP), anti-HSP70 (Proteintech, 10995-1-AP), anti-TSG101 (Proteintech, 28283-1-AP), anti-CD63 (Proteintech, 25682-1-AP), anti-CD44 (Proteintech, 15675-1-AP), anti-EGFR (Cell Signaling Technology, USA, 4267S), anti-IRP1 (Santa Cruz, sc-166022), anti-IRP2 (Santa Cruz, sc-33682), and anti-NRF2 (Santa Cruz, sc-365949).

### Cell proliferation assay

The proliferation of different cell lines was determined using the xCELLigence Real-Time Cell Analyzer (RTCA)-MP system (Acea Biosciences/Roche Applied Science, CA, USA).

### Measurements of the total intracellular iron contents

The total intracellular iron content was determined using an Intracellular Iron Colorimetric Assay Kit (E1042, APPLYGEN) according to the manufacturer's instructions. The absorbance was measured at 550 nm using a microplate reader (PerkinElmer, MA, USA). The total intracellular iron concentration was calculated from the standard curve.

### Ribonuclease R (RNase R) digestion and actinomycin D assays

RNase R (2 U/µg RNA) was added to the extracted RNA. Reverse transcription was performed, and the results were analyzed using qPCR. Actinomycin D (100 ng/mL; Sigma‒Aldrich, MO, USA) was added. Samples were collected at 0, 4, 8, 12, and 24 h for qPCR.

### Subcellular fractionation assays

The assay was performed according to the manufacturer's protocol (Beyotime Biotechnology, China). Briefly, the cell precipitate was lysed using cytoplasmic protein extraction reagent A and nuclear protein extraction reagent B. The internal reference for cytoplasmic proteins was β-actin, and the internal reference for nuclear proteins was NEAT1. qPCR was used to analyze the expression of the target genes in different fractions.

### Intracellular fluorescence *in situ* hybridization (FISH)

A probe designed for circHIF1α was used for *in situ* hybridization, and the assay was performed according to the manufacturer's protocol (RiboBio, China). The sequences of the FISH probes used in our study are shown in **Table S1**.

### Luciferase reporter assay

**Figure 1:**
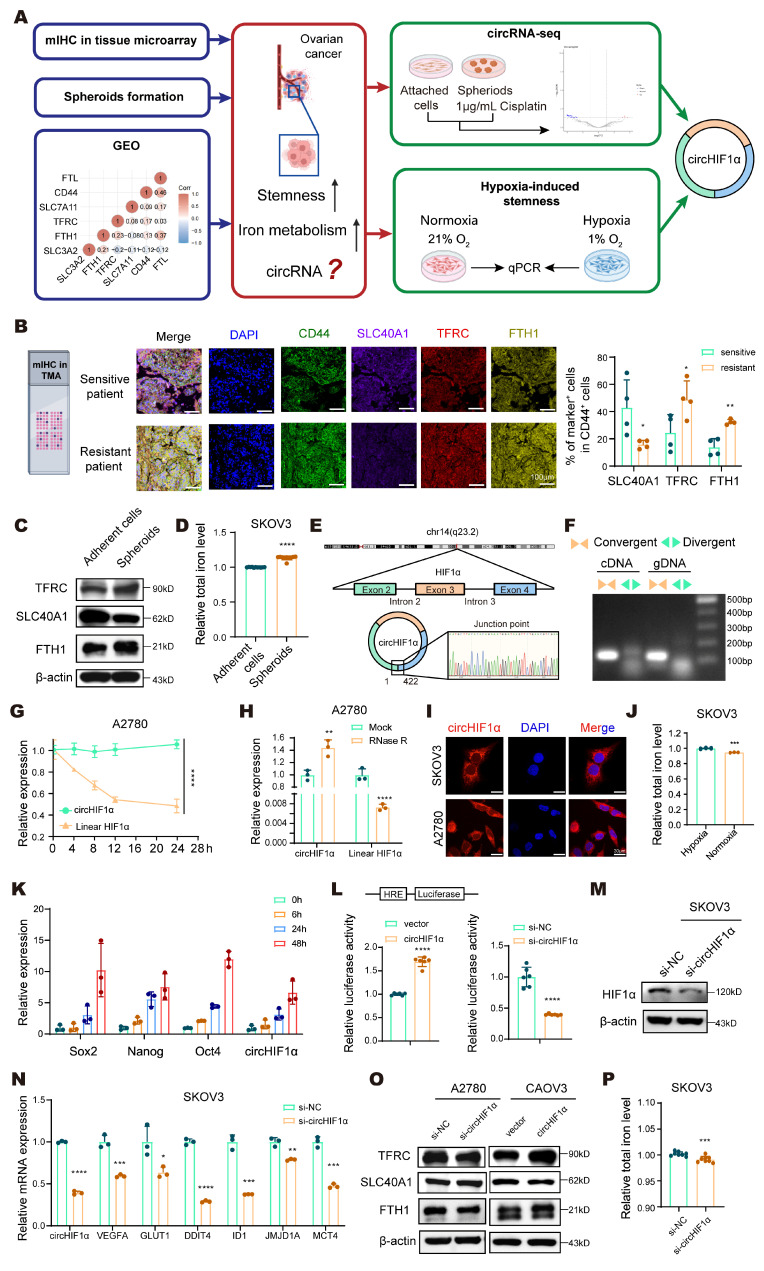
** Hypoxia-induced circHIF1α modulates iron metabolism in ovarian cancer stem-like cells. A**, Workflow for screening circHIF1α from ovarian cancer as a target. The icons of this figure were created using BioRender.com. **B**, Multiplex immunohistochemical (mIHC) staining for DAPI, CD44, SLC40A1, TFRC and FTH1 in the ovarian cancer tissue microarray (TMA). The icons of this figure were created using BioRender.com. Scale bar, 100 µm. **C**, The protein expression of TFRC, SLC40A1, and FTH1 in adherent SKOV3 cells and spheroids. **D**, Total iron levels in adherent SKOV3 cells and spheroids. **E**, Schematic illustration showing the genomic loci of circHIF1α. **F**, PCR was performed to detect circHIF1α expression in cDNA and gDNA from SKOV3 cells using divergent or convergent primers. **G**, The expression of circHIF1α and its linear transcript following actinomycin D (100 ng/mL) treatment. **H**, The expression of circHIF1α and the linear transcript HIF1α in A2780 cells with or without RNase R (2 U/µg RNA) treatment. **I**, Subcellular localization of circHIF1α in SKOV3 and A2780 cells, as determined using fluorescence *in situ* hybridization (FISH). Scale bar, 30 µm. **J**, Total iron levels in SKOV3 cells exposed to hypoxia or normoxia. **K**, Expression levels of Sox2, Nanog, Oct4, and circHIF1α in SKOV3 cells at 0, 6, 24, and 48 h after hypoxia exposure. **L**, HRE-luciferase reporter assays revealed that the overexpression of circHIF1α increased HIF1α transcriptional activity, whereas the knockdown of circHIF1α reduced HIF1α activity. **M**, Expression of HIF1α in SKOV3 cells transfected with si-NC/si-circHIF1α. **N**, Expression levels of VEGFA, GLUT1, DDIT4, ID1, JMJD1A, MCT4 and circHIF1α in SKOV3 cells transfected with si-NC/si-circHIF1α. **O**, Western blot analysis of TFRC, SLC40A1 and FTH1 expression in A2780 cells transfected with si-NC/si-circHIF1α or CAOV3 cells transfected with vector/ circHIF1α. **P**, Total iron levels in SKOV3 cells treated with si-NC/si-circHIF1α. The data are presented as the mean ± SD; *, P < 0.05; **, P < 0.01; ***, P < 0.001; ****, P < 0.0001.

CAOV3 and OVCAR3 cells were seeded in 12-well plates and then transfected with mi-NC/miR-375 mimic, pmirGLO-circHIF1α WT/MUT, or pmirGLO-SLC7A11 3'UTR WT/MUT. A Renilla luciferase plasmid was cotransfected as an internal control. After 48 h, the samples were processed using the Dual Luciferase Reporting and Detection System (Promega, Madison, WI, USA) according to the manufacturer's instructions. As shown in Figure 1L, SKOV3 cells were cotransfected with the HRE-luciferase plasmid and si-NC/si-circHIF1α or vector/circHIF1α. Cells were subjected to hypoxia for 6 h before the luciferase activities of the cell lysates were measured.

### Prussian blue staining

Iron accumulation in tissues was detected using Prussian blue staining (60533ES20, Yeasen, China) according to the manufacturer's instructions.

### Multiplex immunohistochemistry (mIHC)

mIHC staining was performed using a PANO 5-plex immunohistochemistry (IHC) kit (Panovue). Assays were performed using the following antibodies: anti-SLC40A1, anti-TFRC/CD71, anti-FTH1, anti-SLC3A2/CD98, anti-SLC7A11, and anti-CD44. The cell nuclei were stained with DAPI and scanned, and multispectral images were captured using a Vectra® Polaris™ fully automatic quantitative pathological imaging analysis system.

### Tumor spheroid formation assays

After digestion, 5×10^3^ cells were seeded in each well of a low-adherence six-well plate containing DMEM/F12 (Gibco) supplemented with 20 ng/mL EGF, 20 ng/mL bFGF, and 2% B27. After 10-14 days, the quantity and dimensions of the cell spheroids were examined using a microscope.

### Cystine uptake assays

The cystine uptake capacity of the cells was evaluated using a cystine uptake assay kit (Dojindo Molecular Technologies, Inc.). Finally, the fluorescence was detected using a fluorescence microplate reader (PerkinElmer, MA, USA) at λex=490 nm and λem=535 nm.

### Measurement of Glutathione (GSH)/Glutathione Disulfide (GSSG) levels

The cortical GSH content was measured using a GSH and GSSG assay kit (Beyotime Biotechnology, Shanghai, China) according to the manufacturer's instructions. The GSH content was measured using a microplate reader (PerkinElmer, MA, USA; OD =412 nm). The GSH content of the test samples was calculated as follows: total GSH-GSSG × 2.

### Ferroptosis assays

FerroOrange (F374, Dojindo Molecular Technologies Inc.) was used to detect cellular iron. Briefly, cells were seeded on a microscope cover glass in culture dishes, incubated for 24 h, stained with FerroOrange (1 μM) for 30 min at 37 °C, washed, and then observed under an inverted confocal laser scanning microscope. For Liperfluo staining (L248, Dojindo Molecular Technologies Inc.), the cells were stained with Liperfluo (10 μM) for 1.5 h at 37 °C, washed, and then observed under an inverted confocal laser scanning microscope. The malondialdehyde (MDA) content was determined using an MDA detection kit (S0131, Beyotime Biotechnology) according to the manufacturer's instructions. Intracellular glutathione peroxidase 4 (GPX4) activity was measured using a cellular glutathione peroxidase assay kit (S0056, Beyotime Biotechnology).

### Animal experiments

Four-week-old female BALB/c nude mice were purchased from Beijing HFK Bioscience (Beijing, China) and used to establish the subcutaneous or peritoneal metastasis model. sh-NC/sh-circHIF1α-transfected SKOV3 cells were injected at a density of 2×10^6^ cells/mouse. Four-week-old female NOD-SCID mice were purchased from Beijing HFK Biosciences (Beijing, China) and used for the spheroid cell tumorigenicity assay. First, sh-NC/sh-circHIF1α SKOV3 cells were cultured in 6-well low-adhesion plates. Approximately 10-14 days later, spheroids formed in each well. The spheroids were isolated, dissociated into individual cells, and then different numbers of cells were injected into the axilla of each group of NOD/SCID mice. The results were statistically analyzed after six weeks. For the animal model treated combinations of drugs, SKOV3 cells were injected subcutaneously into the left axilla of BALB/c nude mice. After 7 days, cisplatin was injected intraperitoneally, and PBS, cell membrane-coated si-NC biomimetic nanoparticles (CMNP-siNC), or cell membrane-coated si-circHIF1α biomimetic nanoparticles (CMNP-siRNA) were injected through the tail vein.

### Fluorescence *in situ* hybridization (FISH) of tissues

Oligonucleotide probes with FITC labels specifically targeting the circHIF1α junction point (ACTTATCCATTTCTGTGTGTA) were designed and synthesized by GeneSeed (Guangzhou, China). FISH was performed using the circRNA/miRNA Fluorescent *In Situ* Hybridization Test Kit (GeneSeed, Guangzhou, China) according to the manufacturer's guidelines. After blocking, mIHC staining was performed using a PANO 5-plex IHC kit (Panovue). After staining, the cell nuclei were stained with DAPI to locate the cells. Finally, an antifluorescence quenching agent (Panovue) was used for mounting. Scanned and multispectral images were captured using the Vectra Polaris fully automatic quantitative pathological imaging analysis system.

### RNA-binding protein immunoprecipitation (RIP)

The RIP assay was performed using a RIP Kit (GeneSeed, Guangzhou, China) according to the manufacturer's instructions. The assays were performed using the following antibodies: anti-SLC3A2 (Proteintech, 15193-1-AP), anti-IGF2BP3 (Proteintech, 14642-1-AP), anti-m6A for MeRIP (Synaptic Systems, 202003), and anti-AGO2 (Proteintech, 67934-1-Ig).

### Extraction of plasma-derived exosomes

The plasma was centrifuged at 2,000 × g for 20 min and at 10,000 × g for 20 min. The supernatant was removed, and 1/3 the volume of Ribo™ exosome isolation reagent (RiboBio, China) was added to the samples and incubated at 4 °C for 30 min, after which the sample was centrifuged at 15,000 × g for 2 min; the supernatant was aspirated to obtain the exosomes. The long-chain noncoding RNA GM13008 was added as an external parameter. Subsequently, TRIzol was added to extract exosomal RNA.

### Collection and identification of cell-derived exosomes

The collection and identification of cell-derived exosomes were performed as previously described 30. Exosomes were identified by observation under a transmission electron microscope (TEM) and by performing a nanoparticle tracking analysis (NTA) using a NanoSight NS300 instrument (Malvern Instruments Ltd., UK).

### Synthesis of biomembrane-coated nanoparticles

SKOV3 cell membranes were obtained from NCure Co., Ltd. (Wuhan, China). Then, 25 kDa polyethyleneimine (PEI) was mixed with the siRNA at a mass ratio of 0.8:1 (N/P ratio of approximately 10:1). The final CMNP-siRNA nanoparticles were prepared by adding the cell membranes at a mass ratio of 12:0.8:1 (cell membrane: PEI: siRNA) under ultrasonic conditions. Changes in the morphology of CMNP-siRNA were monitored using TEM. The particle size and zeta potential were measured using dynamic light scattering (DLS).

### Cellular uptake and endosome escape

SKOV3 cells were seeded into confocal dishes and cultured overnight. The cells were then incubated with Cy5-labeled siRNA loaded with CMNP-siNC and CMNP-siRNA (Cy5-siRNA: 200 nM) for 2, 4, 6, or 12 h. Afterward, the cells were washed three times with PBS and incubated with LysoTracker Green (40738ES50, YEASON) for 30 min. Finally, the nuclei were stained with Hoechst (KGA1815-1, KeyGEN BioTECH) for 10 min and washed three times with PBS for observation using a confocal microscope.

### *In vitro* cytotoxicity of CMNP-siRNA

SKOV3 cells were seeded in a 96-well plate and cultured overnight. Then, CMNP-siNC or CMNP-siRNA (siRNA concentration: 50, 100, 150, 200, 300 nM) was added, and the cells were incubated for 48 h. A cell counting kit-8 (CCK-8) assay was performed to assess cell viability.

### *In vivo* imaging of CMNP-siRNA

The mice bearing tumors were randomly divided and injected with Cy5-labeled CMNP-siRNA, CMNP-siNC, or free siRNA (dosage: 1.0 mg Cy5-siRNA equiv./kg). At 2, 4, 8, and 24 h after the injection, the mice were anesthetized with isoflurane (RWD Life Science, R510-22-10), and whole-body near-infrared fluorescence images were acquired using a Tanon ABL X5 Live Animal Imaging System. The mice were then euthanized at 24 h postinjection, the major organs (heart, liver, spleen, lung, kidney and tumor) were collected, and the samples were washed. *Ex vivo* imaging of these organs was performed using a Tanon ABL X5 Live Animal Imaging System 31.

### Statistical analysis

The data were analyzed using GraphPad Prism 9 on the Windows platform. A t-test was used to compare the significance of differences between two groups. The chi-square test or Fisher's test was used to explore associations between specific gene expression levels and clinical parameters. A P value < 0.05 was considered statistically significant. The data are presented as the mean ± SD; ns, not significant; *, P < 0.05; **, P < 0.01; ***, P < 0.001; ****, P < 0.0001.

## Results

### Hypoxia-induced circHIF1α modulates iron metabolism in ovarian cancer stem-like cells

Dysregulated iron metabolism is a recognized characteristic of CSCs. However, how stemness specifically influences iron metabolism in ovarian cancer cells remains unclear. We first performed mIHC staining of a tissue microarray (TMA) comprising cisplatin-sensitive and cisplatin-resistant ovarian cancer specimens. Using CD44 as a stemness marker, we assessed the expression of key iron metabolism-related proteins in CD44⁺ cells. The results revealed that in cisplatin-resistant tissues, the proportion of cells positive for SLC40A1 decreased, whereas the proportion of cells positive for TFRC and iron storage protein ferritin heavy chain 1 (FTH1) increased, indicating increased iron dependency in resistant cells (Figure 1A, B). We further compared the expression of iron metabolism markers and total intracellular iron levels between adherent and spheroid cultures of SKOV3 cells. Notably, the iron metabolism of the spheroids was upregulated (Figure 1C), and the cellular iron levels were increased (Figure 1D). An analysis of the GEO database indicated a positive correlation between stemness and iron metabolism in ovarian cancer (Figure 1A). Collectively, these results provide consistent evidence that increased stemness is associated with increased iron metabolism in ovarian cancer.

To investigate the intrinsic link between stemness characteristics and dysregulated iron metabolism in ovarian cancer, we established an *in vitro* stem cell-based drug resistance model. We used SKOV3 cells to enrich cancer stem-like cells and promote spheroid formation. We treated these spheroids with cisplatin at a final concentration of 1 μg/mL for one week after spheroid formation to simulate the clinical setting of drug resistance. Using adherent SKOV3 cells as controls, we subsequently performed circRNA sequencing of the spheroids and attached cells to identify differentially expressed circRNAs (Figure 1A). The results revealed that circHIF1α was highly expressed in the spheroids. circHIF1α is located in the exon region of chromosome 14, derived from exons 2 to 4 of the HIF1α gene, and has a length of 422 nt (Figure 1E). Divergent and convergent primers were designed to specifically amplify the circular and linear isoforms, confirming the circular structure of circHIF1α (Figure 1F). The RNA was further treated with actinomycin D (Figure 1G) and subjected to RNase R digestion (Figure 1H), which demonstrated the pronounced stability of circHIF1α compared with its linear counterpart. In addition, qRT‒PCR using both oligo dT and random primers revealed that circHIF1α lacks a polyA tail, further supporting its noncanonical circular topology (Figure S1A). The qRT‒PCR and FISH results showed that circHIF1α is distributed in both the nucleus and the cytoplasm, mainly in the cytoplasm (Figure 1I; Figure S1B).

We further induced stemness under hypoxic conditions and simultaneously measured the changes in the intracellular iron concentration and expression of circHIF1α (Figure S1C). Under hypoxic conditions, the iron content within the cells increased (Figure 1J). While the expression of stemness markers (Sox2, Nanog, and Oct4) increased, the expression of circHIF1α was also upregulated (Figure 1K). Moreover, under hypoxic conditions, circHIF1α enhanced the transcriptional activity of HIF1α (Figure 1L), increased its expression (Figure 1M), and activated its downstream hypoxia signaling pathway (Figure 1N). Knockdown of circHIF1α resulted in reduced iron metabolism and decreased overall intracellular iron levels (Figure 1O, P). In addition, we investigated whether it affects other regulators of iron metabolism. The results revealed that knockdown of circHIF1α in SKOV3 cells reduced the expression levels of iron regulatory protein (IRP)1, IRP2 and NRF2 (Figure S1D).

### CircHIF1α sustains ovarian cancer stemness and iron homeostasis to promote ferroptosis resistance, chemoresistance, and malignant phenotypes* in vitro*

**Figure 2:**
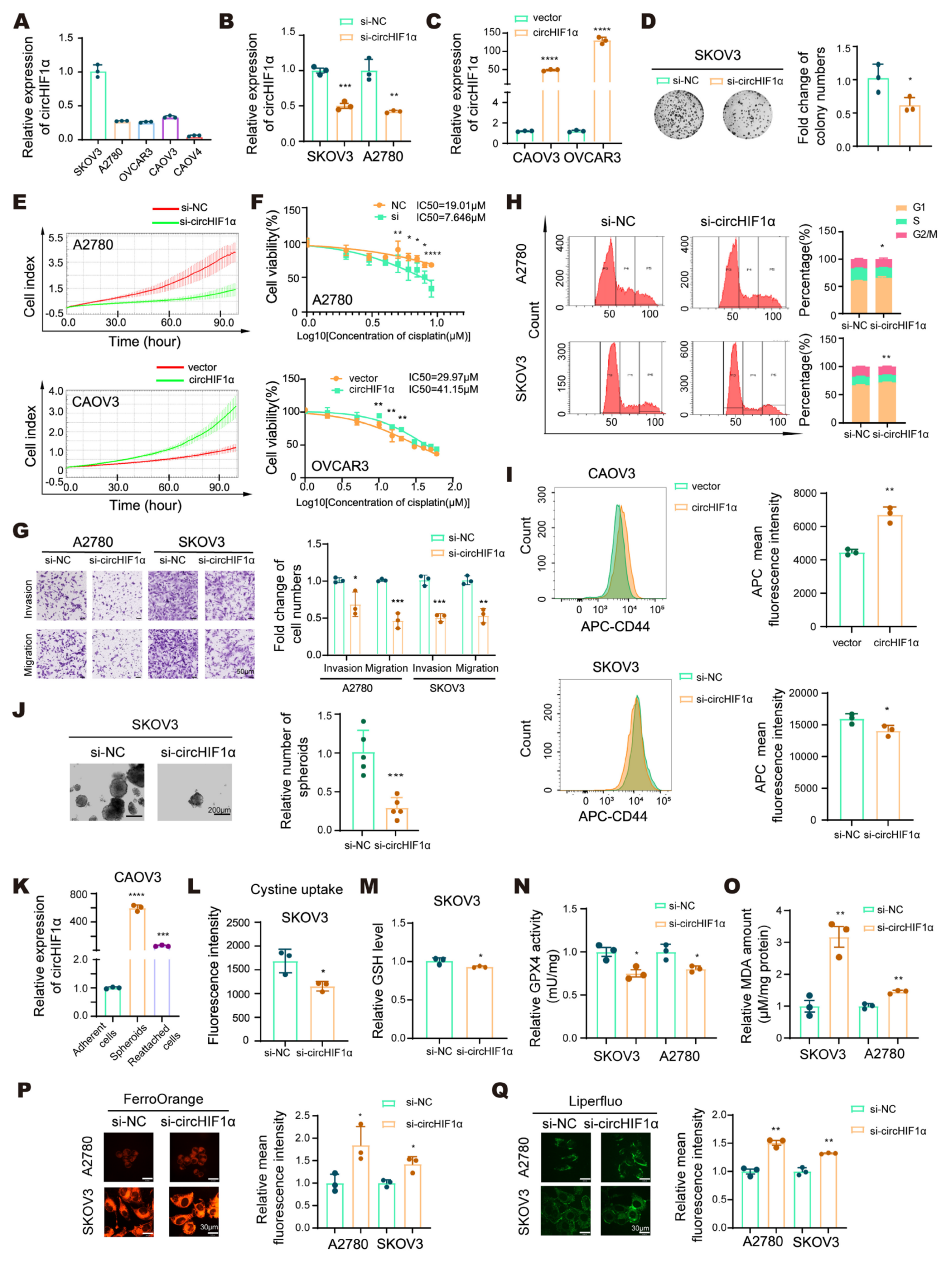
** CircHIF1α sustains ovarian cancer stemness and iron homeostasis to promote ferroptosis resistance, chemoresistance, and malignant phenotypes *in vitro*. A**, qRT‒PCR analysis of circHIF1α levels in epithelial ovarian cancer (EOC) cell lines. **B**, Expression of circHIF1α in SKOV3 and A2780 cells transfected with si-NC/si-circHIF1α. **C**, Expression of circHIF1α in CAOV3 and OVCAR3 cells transfected with vector/circHIF1α. **D**, Cell proliferation was evaluated using colony formation assays. **E**, Growth of A2780 cells transfected with si-NC/si-circHIF1α or CAOV3 cells transfected with vector/circHIF1α using the xCELLigence Real-Time Cell Analyzer (RTCA)-MP system. **F**, A cell counting kit-8 (CCK-8) assay was performed to evaluate the viability of A2780 cells transfected with si-NC/si-circHIF1α or OVCAR3 cells transfected with vector/circHIF1α and then treated with various concentrations of cisplatin. **G**, Transwell assays were used to analyze the invasion and migration of A2780/SKOV3 cells transfected with si-NC/si-circHIF1α. Scale bar, 50 µm. **H**, Flow cytometry was performed to analyze the cell cycle distribution of A2780 and SKOV3 cells transfected with si-NC/si-circHIF1α. The results of the quantitative analyses are presented on the right. **I**, Flow cytometry analysis of CD44 expression levels in spheroids generated from CAOV3 cells transfected with vector/circHIF1α or SKOV3 cells transfected with si-NC/si-circHIF1α.** J**, Representative images of spheroids generated from SKOV3 cells transfected with si-NC/si-circHIF1α. Scale bar, 200 µm. **K**, Relative expression of circHIF1α in adherent CAOV3 cells, spheroids, and spheroid-reattached cells. **L**, Cystine uptake capacity of SKOV3 cells treated with si-NC/si-circHIF1α. **M**, Relative glutathione (GSH) levels in SKOV3 cells transfected with si-NC/si-circHIF1α. **N**, Relative glutathione peroxidase 4 (GPX4) activity in SKOV3/A2780 cells transfected with si-NC/si-circHIF1α. **O**, Relative malondialdehyde (MDA) amount in SKOV3/A2780 cells transfected with si-NC/si-circHIF1α. **P**, Fe^2+^ levels detected using FerroOrange. The relative mean fluorescence intensity is shown on the right. Scale bar, 30 µm. **Q**, Representative images of Liperfluo staining. The relative mean fluorescence intensity is shown on the right. Scale bar, 30 µm. The data are presented as the mean ± SD; *, P < 0.05; **, P < 0.01; ***, P < 0.001; ****, P < 0.0001.

We further investigated the biological functions of circHIF1α by examining its expression levels in a panel of ovarian cancer cell lines to select appropriate models for knockdown and overexpression (Figure 2A), followed by a validation of the transfection efficiency (Figure 2B, C; Figure S2A). Functional assays revealed that knockdown of circHIF1α suppressed colony formation (Figure 2D), proliferation (Figure 2E), cisplatin resistance (Figure 2F), and invasion/migration (Figure 2G) and induced cell cycle arrest (Figure 2H) in ovarian cancer cells. Conversely, the overexpression of circHIF1α increased proliferation, invasion, migration, and cisplatin resistance (Figure 2E, F; Figure S2B-D). These results indicated that circHIF1α plays a cancer-promoting role in ovarian cancer.

Next, we sought to determine the role of circHIF1α in the regulation of CSC-like properties. Flow cytometry analysis indicated increased CD44 expression in the circHIF1α-overexpressing spheroids (Figure 2I). Under low-attachment culture conditions, we found that the spheroid-forming ability was impaired upon the knockdown of circHIF1α in SKOV3 and A2780 cells, whereas it was enhanced following circHIF1α overexpression in OVCAR3 and CAOV3 cells (Figure 2J; Figure S2E-G). Additionally, we measured circHIF1α expression in CAOV3 cells under adherent, spheroid, and reattached conditions. The results showed that circHIF1α levels were higher in spheroids than in adherent cells and decreased upon reattachment (Figure 2K).

Dysregulated iron metabolism, including iron overload, is a key driver of ferroptosis. Despite their high iron demand and accumulation, CSCs exhibit resistance to ferroptosis, suggesting the existence of adaptive mechanisms for maintaining iron homeostasis 32. We investigated whether circHIF1α regulates ferroptosis via iron metabolism by assessing cystine uptake (Figure 2L; Figure S2H), GSH levels (Figure 2M; Figure S2I), GPX4 activity (Figure 2N; Figure S2J), MDA amounts (Figure 2O; Figure S2K), Fe²⁺ levels (Figure 2P), and lipid peroxidation (Figure 2Q). Knockdown of circHIF1α led to reduced cystine uptake, a decreased GSH content, reduced GPX4 activity, increased MDA content and elevated levels of Fe²⁺ and lipid peroxidation, indicating increased susceptibility to ferroptosis. Conversely, the overexpression of circHIF1α increased cellular resistance to ferroptosis.

### Exosome-mediated circHIF1α transfer drives stemness maintenance and cisplatin resistance in ovarian cancer

**Figure 3:**
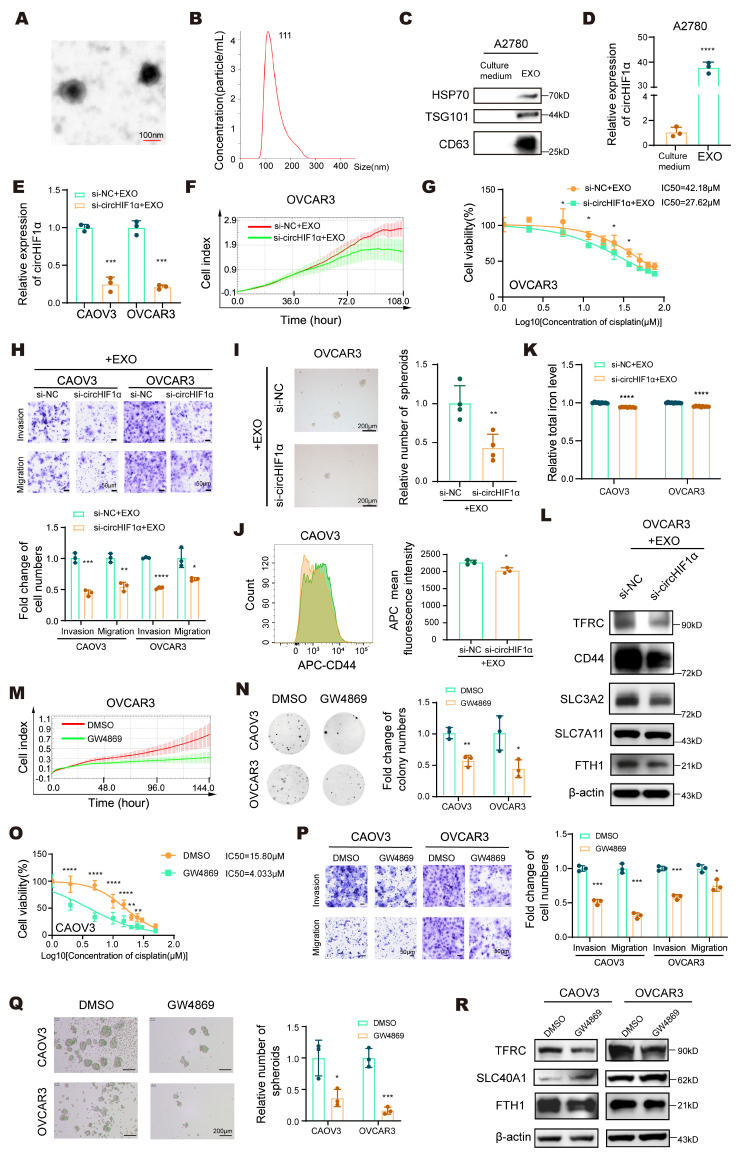
** Exosome-mediated circHIF1α transfer drives stemness maintenance and cisplatin resistance in ovarian cancer. A**, Representative transmission electron microscopy (TEM) images of exosomes derived from A2780 cells. Scale bar, 100 nm. **B**, NanoSight analysis of exosomes derived from A2780 cells. **C**, Western blot analysis of the expression of the exosomal protein markers HSP70, TSG101, and CD63 in exosomes (EXO) and culture medium. **D**, The expression of circHIF1α in culture medium and EXO. **E**, The expression of circHIF1α in CAOV3/OVCAR3 cells treated with si-NC/si-circHIF1α and A2780 exosomes. **F**, Analysis of the growth of OVCAR3 cells treated with si-NC/si-circHIF1α and A2780 exosomes using the xCELLigence RTCA-MP system. **G**, A CCK-8 assay was performed to evaluate the viability of OVCAR3 cells treated with si-NC/si-circHIF1α and A2780 exosomes and further treated with various concentrations of cisplatin. **H**, Transwell assays were performed to analyze the invasion and migration of CAOV3/OVCAR3 cells treated with si-NC/si-circHIF1α and A2780 exosomes. The results of the quantitative analyses are presented in histograms. Scale bar, 50 µm. **I**, Representative images of spheroids generated from OVCAR3 cells treated with si-NC/si-circHIF1α and A2780 exosomes. The results of the quantitative analyses are presented in histograms on the right. Scale bar, 200 µm. **J**, Flow cytometry analysis of CD44 expression levels in spheroids generated from CAOV3 cells treated with si-NC/si-circHIF1α and A2780 exosomes. **K**, Total iron levels in CAOV3/OVCAR3 cells treated with si-NC/si-circHIF1α and A2780 exosomes. **L**, Western blot analysis of CD44, SLC3A2, SLC7A11, TFRC, and FTH1 expression in OVCAR3 cells treated with si-NC/si-circHIF1α and A2780 exosomes. **M**, Curves showing the proliferation of OVCAR3 cells treated with A2780-derived culture medium (DMSO/GW4869). **N**, Colony formation ability of CAOV3/OVCAR3 cells treated with A2780-derived culture medium (DMSO/GW4869). **O**, A CCK-8 assay was performed to evaluate the viability of CAOV3 cells treated with A2780-derived culture medium (DMSO/GW4869) and then treated with various concentrations of cisplatin. **P**, Transwell assays were performed to analyze the invasion and migration of CAOV3/OVCAR3 cells treated with A2780-derived culture medium (DMSO/GW4869). The results of the quantitative analyses are presented in histograms. Scale bar, 50 µm. **Q**, Representative images of spheroids generated from CAOV3/OVCAR3 cells treated with A2780-derived culture medium (DMSO/GW4869). The results of the quantitative analyses are presented in histograms on the right. Scale bar, 200 µm. **R**, Western blot analysis of TFRC, SLC40A1 and FTH1 expression in CAOV3/OVCAR3 cells treated with A2780-derived culture medium (DMSO/GW4869). The data are presented as the mean ± SD; *, P < 0.05; **, P < 0.01; ***, P < 0.001; ****, P < 0.0001.

Accumulating evidence indicates that exosomes contain functional circRNAs, which play critical regulatory roles in various pathophysiological processes 33. Exosomal circRNAs derived from donor cells can act locally or systemically to modulate recipient cell behavior, thereby promoting tumor progression and performing key functions within the tumor microenvironment (TME) 34. We first collected the cell culture medium and characterized exosomes to investigate whether stemness and iron metabolism phenotypes can be transferred between ovarian cancer cells. Transmission electron microscopy (TEM) revealed the presence of lipid bilayer-enclosed cup-shaped vesicles (Figure 3A). Nanoparticle tracking analysis (NTA) confirmed that the extracted particles were enriched and had a diameter of approximately 111 nm, which is consistent with the typical size of exosomes (Figure 3B). Western blot analysis further validated the expression of the exosomal markers HSP70, TSG101, and CD63 (Figure 3C). We then measured the expression of circHIF1α in exosomes derived from A2780 cells using qRT‒PCR (Figure 3D). CAOV3 and OVCAR3 cells were transfected with si-NC or si-circHIF1α and subsequently treated with A2780-derived exosomes to assess the functional transfer of circHIF1α. The results showed that circHIF1α expression was significantly reduced when si-circHIF1α and exosomes were applied together (Figure 3E). Functional assays, including RTCA (Figure 3F; Figure S3A), CCK-8 (Figure 3G; Figure S3B), colony formation (Figure S3C) and transwell assays (Figure 3H), demonstrated that exosome treatment promoted malignant phenotypes, such as proliferation, invasion, migration, and cisplatin resistance in recipient cells. To further examined the effects of exosomal circHIF1α on stemness and iron metabolism in recipient cells, we observed that treatment with both si-circHIF1α and A2780-derived exosomes reduced spheroid formation (Figure 3I; Figure S3D). Flow cytometry analysis revealed decreased expression of the stemness marker CD44 (Figure 3J) and decreased intracellular iron levels (Figure 3K) under the same treatment conditions. Western blot analysis further indicated that exosomal circHIF1α influenced the expression of CD44, SLC3A2, SLC7A11, TFRC and FTH1 in recipient cells (Figure 3L).

Moreover, we used the exosome secretion inhibitor GW4869 to inhibit the production of exosomes in A2780 cells, collected the medium, and used it to treat CAOV3/OVCAR3 cells. Compared with those in the DMSO treatment group, the proliferation (Figure 3M, N; Figure S3E), cisplatin resistance (Figure 3O; Figure S3F), invasion and migration (Figure 3P) of the receptor cells were reduced after GW4869 treatment. Moreover, spheroid formation (Figure 3Q) and the expression of TFRC, SLC40A1, and FTH1 (Figure 3R) changed accordingly. These results further confirmed that blocking exosome secretion can reverse the malignant phenotype, stemness and iron metabolic reprogramming mediated by circHIF1α in ovarian cancer.

### CircHIF1α increases tumor growth, metastasis, stemness-driven tumorigenesis and iron metabolism *in vivo*

**Figure 4:**
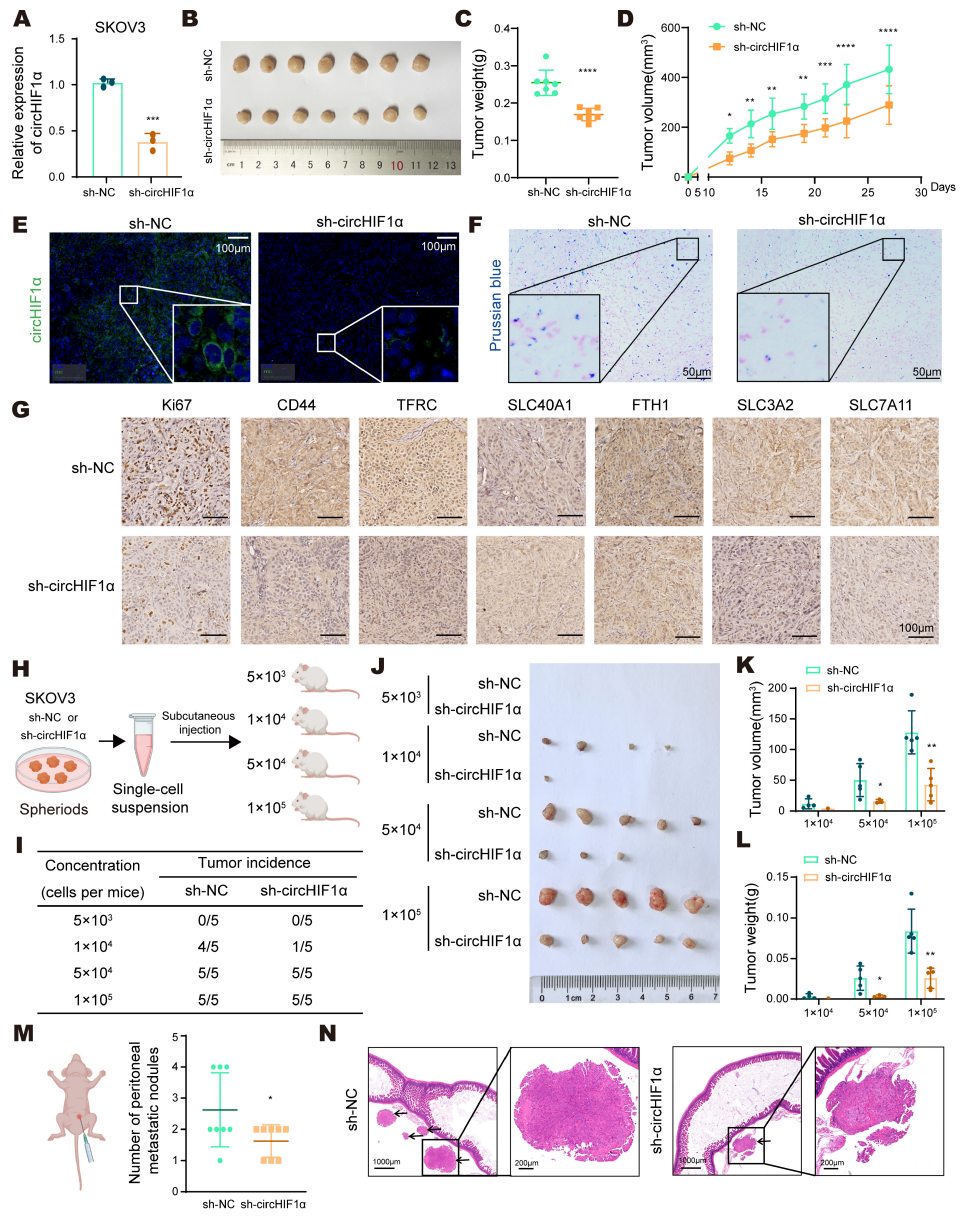
** circHIF1α increases tumor growth, tumor metastasis, stemness-driven tumorigenesis, and iron metabolism *in vivo*. A**, Verification of the stable knockdown of circHIF1α by qRT‒PCR. **B**, The image of xenograft tumors. **C**, Analysis of the growth of tumors in weight. **D**, The tumor volume was measured. **E**, Representative images of FISH of circHIF1α in xenograft tumors. Scale bar, 100 µm. **F**, Representative images of Prussian blue staining for iron in xenograft tumors. Scale bar, 50 µm. **G**, Representative images of immunohistochemical staining for Ki67, CD44, TFRC, SLC40A1, FTH1, SLC3A2, and SLC7A11 in xenograft tumors. Scale bar, 100 µm. **H**, Schematic diagram presenting the experimental procedure of the spheroid cell tumorigenicity assay. The icons of this figure were created using BioRender.com. **I**, The tumor incidence according to the concentration gradient is listed. **J**, Tumor formation by SKOV3 sh-NC or sh-circHIF1α stem-like cells isolated from spheroids. Tumor growth was analyzed in terms of volume (**K**) and weight (**L**). **M**, For the peritoneal metastasis model, four-week-old female BALB/c nude mice were injected intraperitoneally with sh-NC or sh-circHIF1α SKOV3 cells per mouse (n=8). The number of peritoneal metastatic nodules was counted, and the results are presented in a scatter plot. The icons of this figure were created using BioRender.com. **N**, Images of hematoxylin‒eosin (HE) staining of metastatic foci in the abdominal cavity of metastatic tumors from the sh-NC and sh-circHIF1α groups are shown. Scale bars: 1,000 and 200 μm. The data are presented as the mean ± SD; *, P < 0.05; **, P < 0.01; ***, P < 0.001; ****, P < 0.0001.

To evaluate the impact of circHIF1α on ovarian cancer *in vivo*, we established SKOV3 cells stably expressing either a negative control (sh-NC) or circHIF1α-targeting shRNA (sh-circHIF1α). qRT‒PCR confirmed that circHIF1α expression was reduced in sh-circHIF1α cells compared with sh-NC cells (Figure 4A). Using a subcutaneous xenograft model in BALB/c nude mice, we observed that tumors derived from sh-circHIF1α cells exhibited slower growth rates and reduced tumor weight and volume (Figure 4B-D). FISH using a circHIF1α-specific probe further confirmed the decrease in circHIF1α expression in the sh-circHIF1α group (Figure 4E). Prussian blue staining revealed a reduction in the iron content in the sh-circHIF1α group (Figure 4F). Immunohistochemical (IHC) staining showed lower Ki67 labeling, indicating decreased proliferation and reduced expression of CD44, SLC3A2, SLC7A11, TFRC and FTH1. In contrast, the expression of SLC40A1 was increased (Figure 4G).

To investigate the role of circHIF1α in the regulation of CSC-like properties *in vivo*, sh-NC and sh-circHIF1α cells were cultured as spheroids, dissociated into single-cell suspensions, and subcutaneously injected into female NOD/SCID mice at four different cell doses (5×10³, 1×10⁴, 5×10⁴, and 1×10⁵ cells; n=5 mice per group). The knockdown of circHIF1α reduced the tumor incidence (Figure 4H, I), tumor size (Figure 4J, K), and tumor weight (Figure 4L) in mice injected with different numbers of cells. As ovarian cancer frequently develops into intraperitoneal metastasis, we injected sh-NC and sh-circHIF1α cells into the peritoneal cavity of nude mice (n=8 mice per group). Knockdown of circHIF1α led to a decrease in both the number and size of intestinal metastatic nodules (Figure 4M-N). Taken together, these findings indicate that circHIF1α promotes tumor initiation, metastatic potential, and stem-like properties *in vivo*.

### CircHIF1α binds to SLC3A2 and maintains its stability by inhibiting its lysosomal degradation

**Figure 5:**
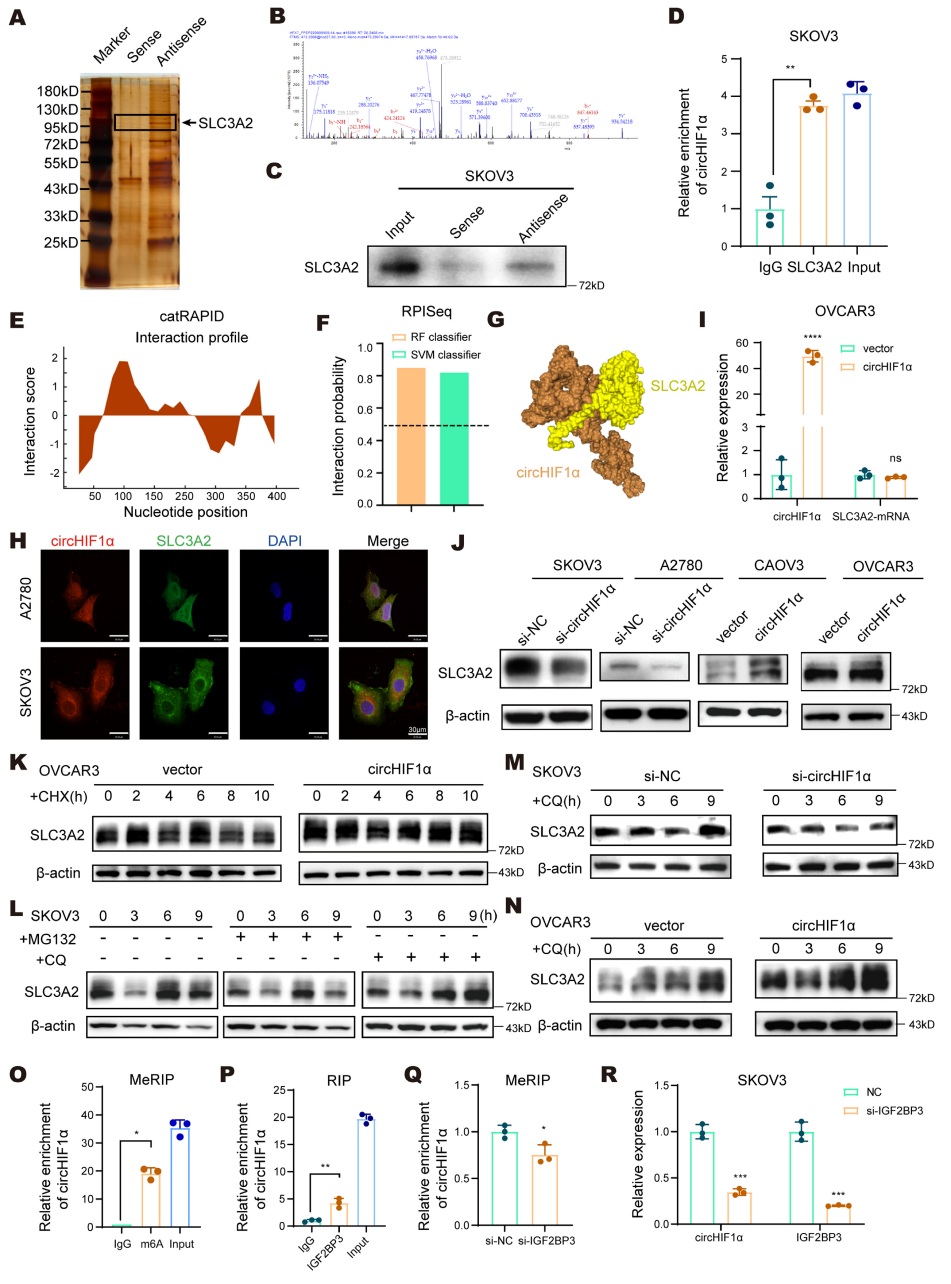
** CircHIF1α binds to SLC3A2 and maintains its stability by inhibiting the lysosomal degradation pathway. A**, Silver staining showed different positive bands in the circHIF1α probe group (Antisense) compared with the control probe group (Sense). Proteins enriched with circHIF1α were identified by liquid chromatography and high-throughput mass spectrometry (LC‒MS/MS). **B**, Mass spectrometry results for the SLC3A2 peptides pulled down by circHIF1α probes. **C**, The interaction between circHIF1α and SLC3A2 in SKOV3 cells was confirmed by Western blotting. **D**, RNA immunoprecipitation (RIP) was performed in SKOV3 cells. **E**, The binding sites between circHIF1α and SLC3A2 were predicted using the catRAPID database. **F**, The possibility of an interaction between circHIF1α and SLC3A2 was detected using the RPISeq database. **G**, Predicted 3D model of the interaction between circHIF1α and SLC3A2. **H**, The colocalization of circHIF1α and SLC3A2 was observed in A2780 and SKOV3 cells. Scale bar, 30 μm. **I,** The expression of the SLC3A2 mRNA in OVCAR3 cells transfected with vector/circHIF1α. **J**, Western blot analysis of SLC3A2 expression in SKOV3/A2780 cells transfected with si-NC/si-circHIF1α or CAOV3/OVCAR3 cells transfected with vector/circHIF1α. **K**, Western blot analysis of SLC3A2 expression in OVCAR3 cells transfected with vector/circHIF1α and treated with cycloheximide (CHX) for 0, 2, 4, 6, 8, 10h. **L**, Western blot analysis of SLC3A2 expression in SKOV3 cells treated with MG132 or chloroquine (CQ) for 0, 3, 6 and 9 h. **M**, Western blot analysis of SLC3A2 expression in SKOV3 cells transfected with si-NC/si-circHIF1α and treated with CQ (10 µM) for 0, 3, 6 and 9 h. **N**, Western blot analysis of SLC3A2 expression in OVCAR3 cells transfected with vector/circHIF1α and treated with CQ (10 µM) for 0, 3, 6 and 9 h. **O**, The MeRIP-qPCR assay verified the m⁶A methylation of circHIF1α. **P**, RIP‒qPCR confirmed the interaction between circHIF1α and IGF2BP3. **Q**, An MeRIP-qPCR analysis was performed with an anti-m⁶A antibody after the knockdown of IGF2BP3. **R**, Expression of circHIF1α and IGF2BP3 in SKOV3 cells transfected with si-NC/si-IGF2BP3. The data are presented as the mean ± SD; ns, not significant; *, P < 0.05; **, P < 0.01; ***, P < 0.001; ****, P < 0.0001.

To further elucidate the molecular mechanism by which circHIF1α functions in ovarian cancer, we designed a probe that targets its back-splice junction (Antisense). RNA pull-down assays were performed in SKOV3 cells using this probe and a control probe (Sense), followed by silver staining and liquid chromatography and high-throughput mass spectrometry (LC‒MS/MS) to identify the differentially bound proteins (Figure 5A). Based on the results, we selected SLC3A2, a protein that is functionally related to circHIF1α and directly associated with ferroptosis, for further investigation (Figure 5B). SLC3A2 serves as a chaperone that stabilizes SLC7A11, and together, they form system Xc⁻, a key cystine/glutamate antiporter. Dysregulation of system Xc⁻ is a well-established trigger of ferroptosis 35. The interaction between circHIF1α and SLC3A2 was validated by RNA pull-down (Figure 5C) and RIP assays (Figure 5D). Bioinformatics analysis using the CatRAPID database (http://s.tartaglialab.com/page/catrapid_group) revealed a strong binding affinity between SLC3A2 and circHIF1α (Figure 5E), and the RPISeq database (http://pridb.gdcb.iastate.edu/RPISeq/) predicted an approximately 80% binding probability (Figure 5F). A predicted 3D interaction model was generated using the HDOCK server (http://hdock.phys.hust.edu.cn/) (Figure 5G) 36. Moreover, immunofluorescence staining of A2780 and SKOV3 cells showed the colocalization of circHIF1α and SLC3A2 (Figure 5H).

Next, we sought to understand how circHIF1α regulates SLC3A2 to confer ferroptosis resistance. PCR analysis revealed that circHIF1α did not affect SLC3A2 mRNA expression (Figure 5I). However, knockdown of circHIF1α reduced SLC3A2 protein levels, whereas its overexpression increased them (Figure 5J), suggesting that circHIF1α may regulate SLC3A2 protein stability. Cycloheximide (CHX) assays confirmed that the overexpression of circHIF1α extended the half-life of the SLC3A2 protein (Figure 5K). Pretreatment with chloroquine (CQ), but not MG132, attenuated SLC3A2 degradation (Figure 5L), indicating that SLC3A2 was primarily degraded via the lysosomal pathway. Consistently, knockdown of circHIF1α promoted the lysosomal degradation of SLC3A2, whereas circHIF1α overexpression suppressed it (Figure 5M-N). Using the circBank online tool (http://www.circbank.cn/index.html#/home), we identified potential m⁶A modification sites on circHIF1α. Mass spectrometry analysis also revealed that the expression of IGF2BP3 (an m⁶A reader) was upregulated in the antisense group. MeRIP-qPCR experiments showed that circHIF1α was indeed subjected to m⁶A methylation (Figure 5O). Furthermore, RIP assays confirmed the direct interaction between circHIF1α and IGF2BP3 (Figure 5P). After the knockdown of IGF2BP3, the level of the m⁶A modification of circHIF1α was reduced (Figure 5Q). Consequently, the stability of circHIF1α decreased, leading to the downregulation of its expression (Figure 5R). Collectively, these results elucidate a regulatory mechanism by which IGF2BP3, an m⁶A reader protein, maintains the stability of circHIF1α and promotes its high expression by recognizing and binding to the m⁶A-modified sites on circHIF1α.

### CircHIF1α acts as an miR-375 sponge to relieve the miR-375-mediated suppression of its target SLC7A11

**Figure 6:**
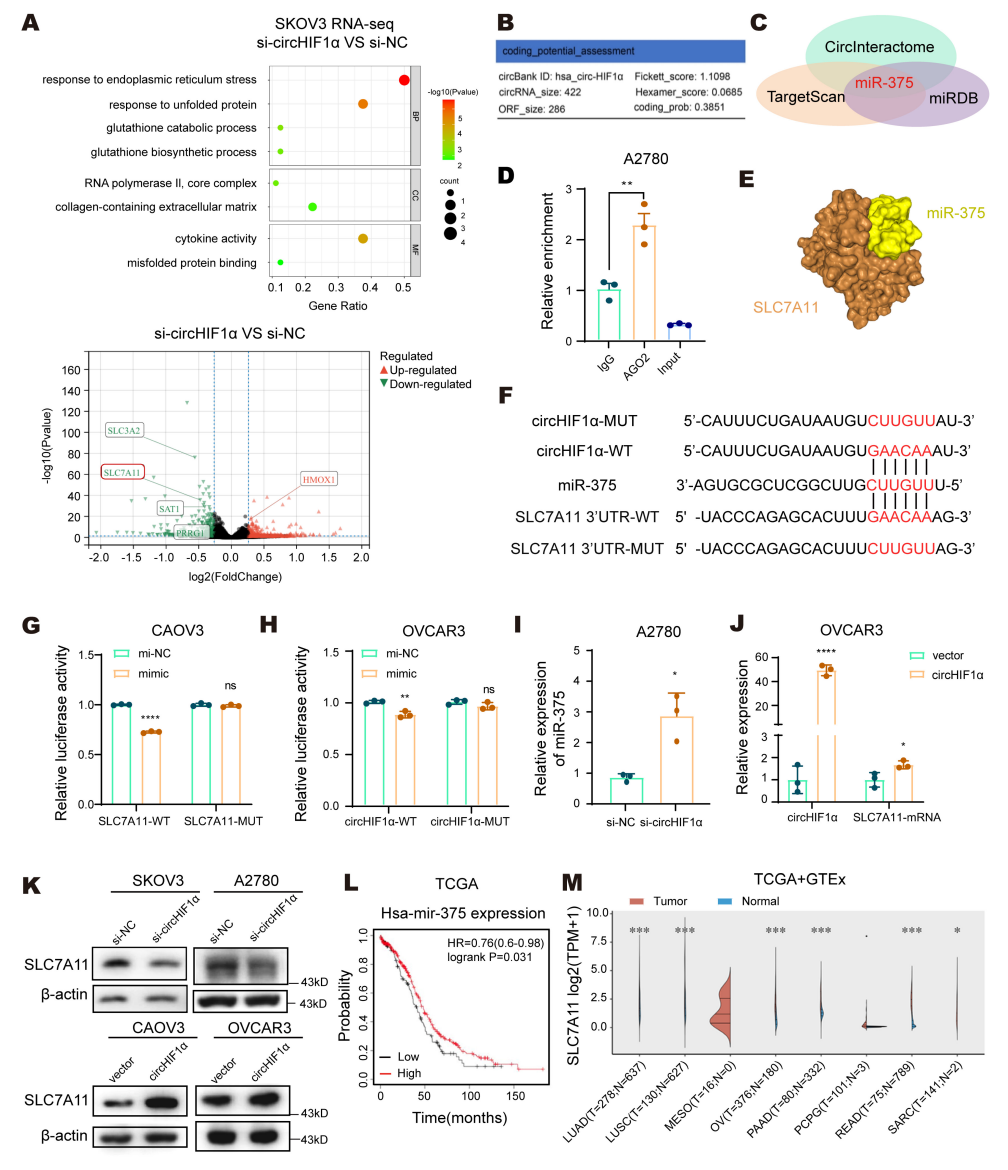
** CircHIF1α acts as an miR-375 sponge to relieve the miR-375-mediated suppression of SLC7A11. A**, Bubble chart showing the functional enrichment pattern of SKOV3 cells transfected with si-NC/si-circHIF1α. The color scale indicates different thresholds of the p value, and the size of the dot indicates the number of genes corresponding to each pathway. BP: biological process; MF: molecular function; CC: cellular component. The bubble chart was plotted using https://www.bioinformatics.com.cn. Volcano plot showing differentially expressed genes in cells transfected with si-circHIF1α compared with those transfected with si-NC. Notable genes are labeled for reference. The volcano plot was drawn using http://www.sangerbox.com/. **B**, The potential of circHIF1α to encode small peptides was predicted using the circBank website. **C**, Venn diagrams of predictions from the CircInteractome, TargetScan and miRDB websites. **D**, RIP assays were conducted in A2780 cells. The enrichment of circHIF1α was determined using qRT‒PCR analysis. **E**, Three-dimensional model of the predicted interaction between miR-375 and SLC7A11. **F**, Schematic diagram of the binding sites between miR-375 and circHIF1α WT/MUT or SLC7A11 3'-UTR WT/MUT. **G**, Luciferase activity was detected in CAOV3 cells cotransfected with SLC7A11 3'-UTR WT/MUT plasmid and miR-375 mimics or mi-NC. **H**, Luciferase activity was detected in OVCAR3 cells cotransfected with circHIF1α WT/MUT plasmid and miR-375 mimics or mi-NC. **I**, Expression of miR-375 in A2780 cells transfected with si-circHIF1α or si-NC. **J**, Expression of circHIF1α and the SLC7A11 mRNA in OVCAR3 cells transfected with vector/circHIF1α. **K**, Western blot analysis of SLC7A11 expression in SKOV3/A2780 cells transfected with si-NC/si-circHIF1α or CAOV3/OVCAR3 cells transfected with vector/circHIF1α. **L**, Kaplan‒Meier survival curves of EOC patients with different miR-375 expression levels obtained from the Kaplan-Meier plotter (http://kmplot.com/analysis). **M**, Expression of SLC7A11 in various tumors and normal tissues in TCGA and GTEx databases. The data are presented as the mean ± SD; ns, not significant; *, P < 0.05; **, P < 0.01; ****, P < 0.0001.

In addition to interacting with proteins, circRNAs can function through multiple mechanisms, such as acting as miRNA sponges or encoding functional peptides. We performed transcriptome sequencing of SKOV3 cells transfected with si-NC or si-circHIF1α to explore the alternative modes by which circHIF1α regulates iron metabolism. The results revealed that genes that were differentially expressed following the knockdown of circHIF1α were significantly enriched in biological processes such as GSH metabolism, GSH biosynthesis, the endoplasmic reticulum stress response, the unfolded protein response, and cytokine activity. Moreover, the level of the SLC7A11 transcript decreased upon knockdown of circHIF1α (Figure 6A). An analysis using the circBank online tool (http://www.circbank.cn/index.html) indicated a low coding potential for circHIF1α (Figure 6B). To assess its potential as a miRNA sponge, we used CircInteractome (https://circinteractome.nia.nih.gov/), TargetScan (https://www.targetscan.org/vert_80/), and miRDB (https://mirdb.org/) to identify overlapping miRNAs and found that miR-375 could be sponged by circHIF1α and directly target the 3'UTR of SLC7A11 (Figure 6C). A RIP assay in A2780 cells confirmed that circHIF1α was enriched in the complex containing the argonaute RISC catalytic component 2 (AGO2) protein (Figure 6D). Furthermore, an analysis using the HDOCK server provided a predicted 3D model of the interaction between miR-375 and SLC7A11 (Figure 6E).

Based on the predicted binding sites of miR-375 with circHIF1α and the SLC7A11 3'UTR, we constructed wild-type (WT) and mutant (MUT) versions of both circHIF1α and the SLC7A11 3'UTR for use in dual-luciferase reporter assays (Figure 6F). The results confirmed that circHIF1α directly binds to miR-375 and that SLC7A11 is a target gene of miR-375 (Figure 6G-H). qRT‒PCR analysis showed that knockdown of circHIF1α in A2780 cells increased miR-375 expression (Figure 6I), whereas overexpression of circHIF1α in OVCAR3 cells increased SLC7A11 mRNA levels (Figure 6J). Western blot analysis indicated that knockdown of circHIF1α reduced SLC7A11 protein levels in SKOV3 and A2780 cells, whereas its overexpression increased SLC7A11 protein levels in CAOV3 and OVCAR3 cells (Figure 6K). Clinically, elevated miR-375 levels in EOC patients were significantly correlated with prolonged overall survival (Figure 6L). An analysis of The Cancer Genome Atlas (TCGA) and Genotype-Tissue Expression (GTEx) data further revealed that SLC7A11 expression was upregulated in multiple tumor types compared with normal tissues (Figure 6M).

### CircHIF1α is upregulated in chemoresistant ovarian cancers and rescues ferroptosis by counteracting Erastin-mediated system Xc⁻ inhibition

**Figure 7:**
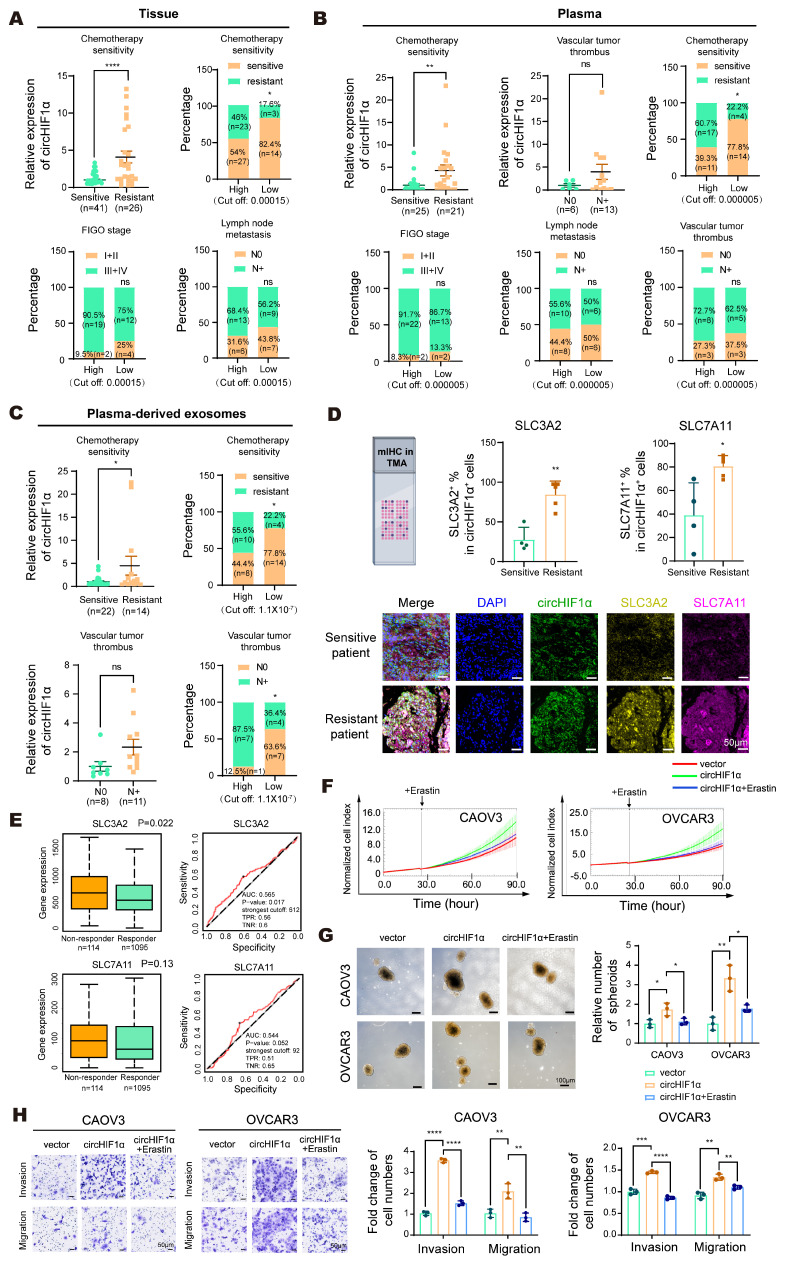
** CircHIF1α is upregulated in chemoresistant ovarian cancers and rescues ferroptosis by counteracting Erastin-mediated system Xc⁻ inhibition. A**, Analysis of circHIF1α expression in ovarian cancer tissues. FIGO: International Federation of Gynecology and Obstetrics (sensitive, n=41; resistant, n=26). **B**, Analysis of circHIF1α expression in plasma samples (sensitive, n=25; resistant, n=21). **C**, Analysis of circHIF1α expression in plasma-derived exosomes (sensitive, n=22; resistant, n=14). **D**, Representative images of mIHC staining of the ovarian cancer tissue microarray (TMA) and the results of quantitative analyses are presented. The icons of this figure were created using BioRender.com. Scale bar, 50 µm. **E**, Expression of SLC3A2 and SLC7A11 in platinum-resistant and platinum-sensitive ovarian cancer tissues (https://rocplot.com/). **F**, Growth of CAOV3 and OVCAR3 cells in response to different treatments (vector, circHIF1α, circHIF1α + Erastin). **G**, Representative images of spheroids generated from CAOV3 and OVCAR3 cells subjected to different treatments (vector, circHIF1α, circHIF1α + Erastin). The results of the quantitative analyses are presented in histograms. Scale bar, 100 μm. **H**, Invasion and migration of CAOV3 and OVCAR3 cells following different treatments (vector, circHIF1α, circHIF1α + Erastin). Representative images of the transwell assay are shown on the left, and the results of the quantitative analyses are presented in histograms. Scale bar, 50 µm. The data are presented as the mean ± SD; ns, not significant; *, P < 0.05; **, P < 0.01; ***, P < 0.001; ****, P < 0.0001.

Next, we evaluated the clinical significance of circHIF1α in ovarian cancer. In tissue samples, circHIF1α expression was higher in chemotherapy-resistant (n=26) patients than in chemotherapy-sensitive (n=41) patients. Stratification by the cut-off value (0.00015) revealed that the proportion of sensitive patients was 82.4% (14/17) in the low-expression group and 54.0% (27/50) in the high-expression group. The proportion of patients with FIGO stage I-II disease was 25.0% (4/16) in the low-expression group and 9.5% (2/21) in the high-expression group. The proportion of patients with N0 (lymph node metastasis-negative) tumors was 43.8% (7/16) in the low-expression group and 31.6% (6/19) in the high-expression group (Figure 7A).

In plasma samples, circHIF1α expression was higher in chemotherapy-resistant patients (n=21) than in chemotherapy-sensitive patients (n=25). Although the difference was not statistically significant, patients with vascular tumor thrombus (N+, n=13) had higher circHIF1α expression than those without thrombus (N0, n=6). Stratification by the predefined cut-off value (0.000005) revealed that the proportion of chemotherapy-sensitive patients was 77.8% (14/18) in the low-expression group and 39.3% (11/28) in the high-expression group. The proportion of patients with FIGO stage I-II disease was 13.3% (2/15) in the low-expression group and 8.3% (2/24) in the high-expression group. The proportion of patients with N0 (lymph node metastasis-negative) tumors was 50.0% (6/12) in the low-expression group and 44.4% (8/18) in the high-expression group. The proportion of patients with N0 (vascular tumor thrombus-negative) tumors was 37.5% (3/8) in the low-expression group and 27.3% (3/11) in the high-expression group (Figure 7B).

In plasma-derived exosomes, circHIF1α expression was higher in chemotherapy-resistant patients (n=14) than in chemotherapy-sensitive patients (n=22). Although a statistically significant difference was not observed, patients with vascular tumor thrombus (N+, n=11) tended to exhibit higher exosomal circHIF1α levels than those without thrombus (N0, n=8). Stratification by the cut-off value (1.1×10⁻⁷) revealed that the proportion of chemotherapy-sensitive patients was 77.8% (14/18) in the low-expression group and 44.4% (8/18) in the high-expression group. The proportion of patients with an N0 (vascular tumor thrombus-negative) status was 63.6% (7/11) in the low-expression group and 12.5% (1/8) in the high-expression group (Figure 7C). Collectively, these findings indicate that circHIF1α is upregulated in patients with chemotherapy-resistant ovarian cancer and is correlated with a poor prognosis. Consistent with these observations, mIHC staining of ovarian cancer tissue microarrays revealed increased circHIF1α expression in chemoresistant patients, with circHIF1α^+^ cells showing increased positivity for both SLC3A2 and SLC7A11 (Figure 7D). An analysis using the ROC plotter database (https://rocplot.com/) further confirmed that SLC3A2 and SLC7A11 were upregulated in patients with platinum-resistant ovarian cancer (Figure 7E). We performed rescue experiments using Erastin, a compound that inhibits system Xc⁻-mediated cystine transport by directly binding to the SLC7A11-SLC3A2 complex, to functionally validate these findings. This binding blocks cystine uptake, depletes intracellular GSH, and induces ferroptosis through excessive lipid peroxidation 37. Our results demonstrated that Erastin treatment reversed the malignant phenotypes promoted by circHIF1α, including increased proliferation (Figure 7F), spheroid formation (Figure 7G), invasion and migration (Figure 7H).

### Biomembrane-coated circHIF1α siRNA-loaded nanoparticles for the precision therapy of ovarian cancer

**Figure 8:**
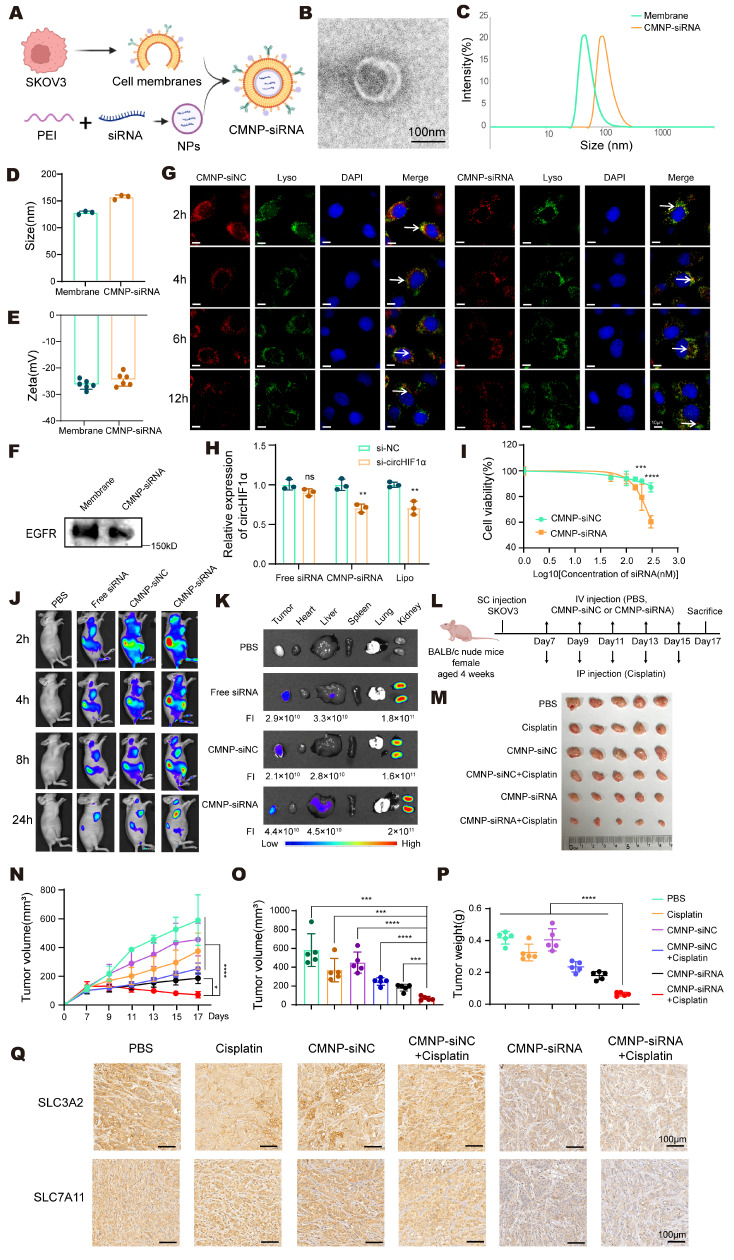
** Biomembrane-coated circHIF1α siRNA-loaded nanoparticles for the precision therapy of ovarian cancer. A**, Schematic diagram of the synthesis of biomembrane-coated nanoparticles. The icons of this figure were created using BioRender.com. **B**, Morphology of cell membrane-coated si-circHIF1α biomimetic nanoparticles (CMNP-siRNA) observed using TEM. Scale bar, 100 nm. **C‒E,** Size and zeta potential of cancer cell membrane (CCM) and CMNP-siRNA determined using dynamic light scattering (DLS). **F**, Western blot of EGFR levels in the cancer cell membrane and CMNP-siRNA. **G**, Representative confocal microscopy images captured after 2, 4, 6, and 12 h of incubation with CMNP-siNC and CMNP-siRNA in SKOV3 cell cultures. The lysosomes were labeled with LysoTracker. Scale bar, 10 μm. **H**, qRT‒PCR analysis of the efficiency of circHIF1α knockdown in cells after an incubation with free siRNA, CMNP-siRNA and Lipofectamine 2000. **I**, CCK-8 assay assessing cell viability after SKOV3 cells were incubated with varying concentrations (50, 100, 150, 200 and 300 nM) of CMNP-siNC or CMNP-siRNA. **J**, *In vivo* whole-body fluorescence images of SKOV3 tumor-bearing nude mice at different time points following the injection of Cy5-labeled CMNP-siRNA, CMNP-siNC, or free siRNA. **K**, *Ex vivo* images of major organs from nude mice at 24 h after the intravenous injection of Cy5-labeled CMNP-siRNA, CMNP-siNC, or free siRNA. FI: fluorescence intensity. **L**, Schematic representation of the animal model treated with the drug combination. The icons of this figure were created using BioRender.com. SC: subcutaneous; IV: intravenous; IP: intraperitoneal. **M**, Representative images of the tumors after the treatments were administered (n=5). **N**, Growth curves of tumors after the treatments were administered (n=5). **O**, Analysis of the tumor volume (n=5). **P**, Analysis of the tumor weight (n=5). **Q**, Representative images of immunohistochemical staining for SLC3A2 and SLC7A11. Scale bar, 100 µm. The data are presented as the mean ± SD; ns, not significant; *, P < 0.05; **, P < 0.01; ***, P < 0.001; ****, P < 0.0001.

To enable efficient targeting of circHIF1α *in vivo*, we developed cell membrane-coated si-NC biomimetic nanoparticles (CMNP-siNC) and cell membrane-coated si-circHIF1α biomimetic nanoparticles (CMNP-siRNA). This system was prepared by complexing 25 kDa polyethylenimine (PEI) with the siRNA at an N/P ratio of 10:1, followed by coating with cell membranes through ultrasonication at a mass ratio of 12:0.8:1 (cell membrane: PEI: siRNA) (Figure 8A). TEM images revealed a distinct membrane bilayer structure surrounding the nanoparticles (Figure 8B). The CMNP-siRNA particles had a diameter of 155.67 ± 3.44 nm (Figure 8C-D) and a negatively charged surface potential of -24.73 ± 2.34 mV (Figure 8E) and expressed EGFR, a characteristic membrane protein of SKOV3 cells (Figure 8F). After the SKOV3 cells were incubated with the nanoparticles for 2, 4, 6, and 12 h, fluorescence microscopy showed that the nanoparticles were primarily localized in the cytoplasm and colocalized with the lysosomes at 2 h. With prolonged incubation, the nanoparticles progressively escaped from the lysosomes (Figure 8G). qRT‒PCR analysis confirmed that compared with CMNP-siNC, CMNP-siRNA significantly downregulated circHIF1α expression in SKOV3 cells (Figure 8H). Furthermore, compared with the nonspecific CMNP-siNC control, CMNP-siRNA effectively killed SKOV3 cells in a concentration-dependent manner, with significantly higher cytotoxicity under the same conditions (Figure 8I).

To monitor the *in vivo* distribution and tumor accumulation of nanoparticles, SKOV3 tumor-bearing mice were intravenously injected with Cy5-labeled free siRNA or nanoparticles. *In vivo* imaging revealed the widespread systemic distribution of both free siRNA and nanoparticles at 2 h postinjection. After 24 h, the signals were largely concentrated in the tumor region. Compared with the free siRNA group, the CMNP-siRNA group exhibited significantly stronger fluorescence intensity in tumors, whereas no signal was detected in the PBS control group (Figure 8J). *Ex vivo* imaging of harvested organs at 24 h showed that Cy5 fluorescence was primarily distributed in the tumors, kidneys, and liver. The fluorescence signal in tumors from the CMNP-siRNA group was stronger than that in the free siRNA group, indicating the *in vivo* targeting ability of the CMNP-siRNA formulation (Figure 8K).

SKOV3 xenograft-bearing nude mice were randomized into groups and treated beginning on the 7th day after inoculation to evaluate the antitumor efficacy of CMNP-siRNA *in vivo*. The mice received intravenous injections of PBS, CMNP-siNC, or CMNP-siRNA every other day. Additionally, cisplatin was administered intraperitoneally (Figure 8L). These results demonstrated that compared with single-agent treatment, combined treatment with cisplatin and CMNP-siRNA achieved superior antitumor efficacy, as reflected by the significant inhibition of tumor growth (Figure 8M-O) and reduced tumor weight (Figure 8P). Notably, the CMNP-siNC + cisplatin control group showed only a slight increase in cisplatin efficacy, which was significantly weaker than the robust antitumor activity observed in the CMNP-siRNA + cisplatin group. These results confirm that the synergistic effect of combination therapy is specifically mediated by circHIF1α silencing rather than a nonspecific effect of the CMNP nanocarrier. IHC staining showed lower expression of SLC3A2 and SLC7A11 in the CMNP-siRNA and CMNP-siRNA + cisplatin groups than in the other groups (Figure 8Q). Representative images of hematoxylin-eosin (HE) staining of the major organs from different groups are presented to evaluate the systemic biosafety of CMNP-siNC/CMNP-siRNA (Figure S4). In summary, these findings indicate that CMNP-siRNA nanoparticles exhibit potent antitumor activity and considerable potential for reversing cisplatin resistance when used in combination with cisplatin.

## Discussion

Recurrence and chemoresistance represent major challenges in the clinical treatment of ovarian cancer 38,39, and CSCs play a pivotal role in this process. Studies have shown that CSCs maintain their stemness and promote tumor progression through unique metabolic reprogramming, among which dysregulated iron metabolism is particularly important 40. This study revealed that under hypoxia-induced stemness conditions, circHIF1α is significantly upregulated in chemoresistant EOC and regulates system Xc⁻ activity through a dual mechanism: it directly binds to the SLC3A2 protein to maintain its stability while simultaneously acting as a sponge for miR-375 to relieve its inhibitory effect on SLC7A11. This dual mechanism collectively increases intracellular cystine uptake and iron metabolism, ultimately conferring resistance to ferroptosis and cisplatin. Based on these findings, we developed cell membrane-coated siRNA nanoparticles targeting circHIF1α. *In vivo* experiments confirmed that these nanoparticles, when combined with cisplatin, significantly inhibited tumor growth and thereby represent a novel therapeutic strategy to overcome chemoresistance.

CSCs exhibit distinct iron metabolism abnormalities, and this metabolic rewiring plays a critical role in maintaining stemness and driving malignant progression. As a core regulator of cellular physiological functions, iron is indispensable for multiple fundamental biological processes, including hemoglobin biosynthesis, cellular energy metabolism, and DNA replication and repair 41. At the molecular level, CSCs establish a sophisticated iron homeostasis system: TFRC-mediated iron uptake, ferritin (composed of FTH/FTL) for iron storage, and SLC40A1-regulated iron efflux work in concert to maintain a dynamic balance 42. This iron metabolic network is finely regulated at multiple levels: IRP1 and IRP2 regulate downstream proteins involved in iron metabolism, including TFRC, SLC40A1 and FTH1 43; transcription of key factors including NRF2, BACH1, and YAP1 form a complex regulatory network. Specifically, NRF2 upregulates FTH, SLC40A1, and HMOX1 to increase antioxidant defenses 44; BACH1 suppresses FTH/FTL and SLC40A1 to promote ferroptosis 45; and YAP1 restricts iron uptake by inhibiting TFRC expression 46. This “iron-dependent” characteristic not only reveals the metabolic vulnerability of CSCs but also provides a theoretical foundation and potential therapeutic targets for anticancer strategies aimed at iron metabolism 27. Nevertheless, the regulatory roles of noncoding ncRNAs in iron metabolism remain largely unexplored. In this study, we discovered that knockdown of circHIF1α reduced the expression levels of the IRP1, IRP2, and NRF2 proteins. These findings indicate that circHIF1α may regulate the expression of IRP1, IRP2 and NRF2 through various mechanisms, such as the competing endogenous RNA (ceRNA) mechanism, RNA-binding protein (RBP)-mediated mRNA stability, and the regulation of the HIF1α pathway. It collaboratively regulates iron metabolism and redox homeostasis in ovarian cancer stem cells.

Hypoxia activates HIF1α signaling, which not only increases glycolysis and angiogenesis but also upregulates the expression of stemness-related genes such as Oct4 and Nanog. This process acts synergistically with the Notch and Wnt pathways to reinforce the self-renewal capacity of CSCs 47. Using high-throughput sequencing and hypoxia-induced models, we identified circHIF1α as a key circRNA that uniquely links platinum resistance and iron metabolism in ovarian cancer stem cells. These findings molecularly connect the three critical pathways: stemness maintenance, chemotherapy resistance, and iron homeostasis. Notably, iron metabolic remodeling in stem-like cells exerts multidimensional control over TME homeostasis and tumor progression. Stem-like cells, including CSCs and premalignant stem cells, dynamically modulate iron acquisition, storage, transport, and utilization as an adaptive response to microenvironmental stress. This reprogramming not only directly influences stem cell survival and function but also interacts with other TME components, such as immune cells, stromal cells, and vascular endothelial cells, to shape a microenvironment conducive to tumor growth, metastasis, and therapeutic resistance. A breakthrough finding of our study is that exosomes derived from ovarian cancer cells that are enriched with circHIF1α induce the dysregulation of iron metabolism in recipient cells, thereby enhancing stemness and cisplatin resistance. This discovery provides new insights into the dynamic regulation of TME homeostasis and, more importantly, reveals a circHIF1α-mediated regulatory network that connects iron metabolism, stemness, and drug resistance. These results highlight circHIF1α as a potential therapeutic target and open new avenues for developing targeted interventions to improve treatment outcomes in patients with ovarian cancer.

Its unique circular structure and sequence characteristics enable circRNAs to perform multiple biological functions, including acting as ceRNAs to sequester miRNAs and post-transcriptionally regulate gene expression by interacting with specific proteins as molecular decoys or functional inhibitors, and, in some cases, encoding functional peptides that participate in tumor-related signaling pathways 48. The system Xc⁻-mediated GSH synthesis pathway provides crucial antioxidant protection 49. Previous studies have established the circRNA-miRNA-SLC7A11 axis as a mechanism that regulates ferroptosis in tumors. For instance, in esophageal cancer, circPVT1 upregulates SLC7A11, FZD3, and GPX4 expression via miR-30a-5p to increase ferroptosis resistance 50. However, research on the circRNA-mediated regulation of SLC3A2, the other critical subunit of system Xc⁻, remains extremely limited. Only one study in an atherosclerosis model reported that circBTBD7-420aa, encoded by hsa_circ_0000563, inhibited abnormal vascular smooth muscle cell proliferation by promoting the K48-linked ubiquitination and degradation of SLC3A2 51. Our study revealed that in ovarian cancer, circHIF1α regulates system Xc⁻ activity through a dual mechanism: it directly binds to the SLC3A2 protein and blocks its lysosomal degradation (rather than ubiquitination) to increase its stability, while simultaneously acting as a sponge for miR-375 to relieve its repression of the SLC7A11 mRNA, thereby synergistically upregulating the expression of both core components of system Xc⁻. This discovery not only expands the understanding of SLC3A2 degradation mechanisms but also provides the first evidence that a single circRNA can simultaneously target both subunits of system Xc⁻ to modulate its function, suggesting that the selective activation of signaling pathways in different microenvironments may lead to distinct regulatory modes.

Efficient targeted drug delivery remains a major bottleneck for disease treatment. As macromolecules carry a negative charge, nucleic acids face multiple biological barriers: they struggle to traverse the negatively charged lipid bilayer of cell membranes, are susceptible to degradation by RNases in plasma and tissues, undergo rapid clearance by the liver and kidneys, and risk recognition by the immune system. Even upon cellular internalization, they often become trapped in endosomal compartments and fail to reach their functional sites. Therefore, the development of efficient, safe, and precise targeted delivery systems is crucial for the development of nucleic acid therapeutics 52,53. Over the past few decades, various nanocarrier-based drug delivery systems have been designed and synthesized to increase therapeutic efficacy 54. Liposomes are the most clinically approved category of drugs. However, their clinical translation has encountered challenges, such as low bioavailability, rapid blood clearance, and induction of innate immune responses. CCMs which are known for their low immunogenicity and homotypic targeting ability, have emerged as promising biomaterials for nanoparticle coatings 55,56. In this study, CCM-coated siRNA nanoparticles exhibited excellent cellular uptake, efficient circHIF1α silencing, and significant antitumor activity both *in vitro* and *in vivo*. These biomimetic nanoparticles offer several distinct advantages: 1) the SKOV3-derived EOC cell membrane coating enables homologous targeting through specific recognition by surface proteins 31; 2) the PEI component not only condenses nucleic acids into stable nanoparticles, preventing aggregation and degradation while maintaining good colloidal stability, but also facilitates endosomal escape via the “proton sponge effect” under acidic conditions in lysosomes, thereby enhancing intracellular delivery 57; and 3) the nanoparticles mediate the specific and efficient knockdown of circHIF1α, directly inhibiting the tumorigenicity of SKOV3 cells in subcutaneous models, and exert superior therapeutic effects when combined with cisplatin.

The low immunogenicity of membrane-coated nanoparticles stems mainly from the biological membrane modification on their surface. This modification can simulate the surface characteristics of cells, thereby reducing the recognition and clearance of nanoparticles by the host immune system 58. However, potential immunological challenges still cannot be completely ruled out. The mononuclear-phagocytic system (MPS, including macrophages in the liver and spleen and dendritic cells) may still recognize and phagocytose nanoparticles to a certain extent, thereby reducing the bioavailability of the nanoparticle carriers and affecting their efficiency in tumor-targeted delivery 59. However, future studies could focus on several optimization strategies to further improve biosafety and reduce potential immune risks, such as modifying the membrane surface with immunosuppressive molecules (e.g., CD47) to evade MPS clearance and adjusting the administration route and frequency to reduce exposure to the immune system. These optimization strategies not only increase the *in vivo* stability and tumor-targeting properties of membrane-coated nanoparticles but also lay the foundation for their future clinical application in the treatment of ovarian cancer through the use of the patient's cell membranes to prepare individualized membrane-coated nanoparticles and achieve precise treatment.

However, this study has several limitations. First, while we focused on the effects of tumor cell-derived exosomal circHIF1α on homologous cancer cells, its potential effects on other components of the TME, such as immune cells and stromal cells, remain unexplored. For example, it may be transported to cancer-associated fibroblasts (CAFs) through exosomes, where it can induce ferroptosis resistance, enabling their sustained activation and increased secretion of protumorigenic factors under chemotherapy stress, indirectly promoting CSC stemness and drug resistance; on the other hand, given the close link between iron metabolic reprogramming and the M2 phenotype 60, circHIF1α-mediated ferroptosis resistance may facilitate M2 macrophage polarization. Ferroptosis-resistant M2 macrophages would accumulate in the TME, release immunosuppressive cytokines, and impair antitumor immunity, further exacerbating chemoresistance. However, the aforementioned analysis is based on logical reasoning and reasonable speculation from existing research, and the actual mechanism may be more complex. Further experimental studies are needed to verify these proposed outcomes. Second, although we observed a clear association between circHIF1α and cisplatin resistance, further validation using dedicated resistant cell lines and *in vivo* resistance models is needed. Furthermore, our data demonstrated that circHIF1α upregulates HIF1α protein expression and enhances its transcriptional activity, thereby activating downstream hypoxic signaling pathways in ovarian cancer cells. Therefore, whether circHIF1α promotes ovarian cancer progression through other molecular mechanisms, such as HIF1α-mediated activation of hypoxic signaling, in addition to the Xc⁻ system, deserves further exploration. The therapeutic efficacy of the biomimetic siRNA nanoparticle system developed in this study requires a systematic evaluation in more clinically relevant models, such as patient-derived organoids (PDOs) and patient-derived xenografts (PDXs), and ultimately in clinical trials.

In summary, our study revealed that circHIF1α not only regulates iron metabolism and stemness by maintaining system Xc⁻ stability but also transmits pro-malignant signals between tumor cells via exosomes. These findings suggest that exosomal circHIF1α is a potential diagnostic biomarker of ovarian cancer. Moreover, we developed a biomimetic nanodelivery system targeting circHIF1α, whose significant tumor-suppressive efficacy opens a new avenue for therapeutic intervention. Future studies should validate the translational value of these findings in more comprehensive experimental systems and further elucidate the multidimensional regulatory network governed by circHIF1α.

## Supplementary Material

Supplementary figures and table.

## Data Availability

The data presented in the current study are available from the corresponding author upon request.

## Abbreviations

AGO2: argonaute RISC catalytic component 2
BP: biological process
CAFs: cancer-associated fibroblasts
CC: cellular component
CCK-8: cell counting kit-8
CCMs: cancer cell membranes
ceRNAs: competing endogenous RNAs
CHX: cycloheximide
circRNAs: circular RNAs
CMNP-siNC: cell membrane-coated si-NC biomimetic nanoparticles
CMNP-siRNA: cell membrane-coated si-circHIF1α biomimetic nanoparticles
CQ: chloroquine
CSCs: cancer stem cells
DLS: dynamic light scattering
DMEM: Dulbecco's modified Eagle's medium
EOC: epithelial ovarian cancer
EXO: exosomes
FBS: fetal bovine serum
FI: fluorescence intensity
FIGO: International Federation of Gynecology and Obstetrics
FISH: fluorescence *in situ* hybridization
FTH1: ferritin heavy chain 1
GPX4: glutathione peroxidase 4
GSH: glutathione
GSSG: glutathione disulfide
GTEx: Genotype-Tissue Expression
HE: hematoxylin-eosin
HIF1α: hypoxia-inducible factor 1 subunit alpha
IC50: half maximal inhibitory concentration
IP: intraperitoneal
IRP: iron regulatory protein
IV: intravenous
LC‒MS/MS: liquid chromatography and high-throughput mass spectrometry
MDA: malondialdehyde
MF: molecular function
miRNAs: microRNAs
mIHC: multiplex immunohistochemistry
MPS: mononuclear phagocyte system
MUT: mutant versions
NTA: nanoparticle tracking analysis
Oct4: POU class 5 homeobox 1
PDAC: pancreatic ductal adenocarcinoma
PDOs: patient-derived organoids
PDXs: patient-derived xenografts
PEI: polyethyleneimine
qRT-PCR: quantitative real-time polymerase chain reaction
RBPs: RNA-binding proteins
RIP: RNA immunoprecipitation
RPMI-1640: Roswell Park Memorial Institute-1640
RTCA: real-time cell analyzer
SC: subcutaneous
sh-circHIF1α: circHIF1α-targeting short hairpin RNA
sh-NC: negative control short hairpin RNA
si-circHIF1α: circHIF1α-targeting small interfering RNA
si-NC: negative control small interfering RNA
SLC40A1: solute carrier family 40 member 1
Sox2: SRY-box transcription factor 2
TCGA: The Cancer Genome Atlas
TEM: transmission electron microscope
TFRC: transferrin receptor
TMA: tissue microarray
TME: tumor microenvironment
WT: wild type
