# Supplementary material for: Biomembrane-coated Nanoparticles Targeting circHIF1α Suppress Ovarian Cancer Metastasis and Cisplatin Resistance by Mediating System Xc⁻ Inactivation via SLC7A11/SLC3A2 to Induce Ferroptosis in Cancer Stem Cells
Source: Int J Biol Sci. 2026 May 18;22(10):5525–47. doi: 10.7150/ijbs.130412 (PMC13215457; doi:10.7150/ijbs.130412)
Supplement: Supplementary file 1 — Supplementary figures and table. [file ijbsv22p5525s1.pdf]

**Supplementary materials**

# Biomembrane-coated Nanoparticles Targeting circHIF1α Suppress Ovarian Cancer Metastasis and Cisplatin Resistance by Mediating System $Xc^-$ Inactivation via SLC7A11/SLC3A2 to Induce Ferroptosis in Cancer Stem Cells

Yinyi Chang[1#], Jingjing Wang[2#], Liying Ma[1], Yi Liu[2], Yumeng Zhu[5], Yu Yi[4], Dongdong Zhang[3]*, Zitong Zhao[1]*, Li Sun[2]*, Yongmei Song[1]*

1. State Key Laboratory of Molecular Oncology, National Cancer Center/National Clinical Research Center for Cancer/Cancer Hospital, Chinese Academy of Medical Sciences and Peking Union Medical College, Beijing, 100021, China.

2. National Cancer Center/National Clinical Research Center for Cancer/Cancer Hospital & Shenzhen Hospital, Chinese Academy of Medical Sciences and Peking Union Medical College, Shenzhen, 518116, China.

3. State Key Laboratory of Epigenetic Regulation and Intervention, Institute of Biophysics, Chinese Academy of Sciences, Beijing, 100101, China.

4. CAS Center for Excellence in Nanoscience, CAS Key Laboratory for Biomedical Effects of Nanomaterials and Nanosafety, National Center for Nanoscience and Technology (NCNST), Beijing, 100190, China.

5. College of Arts & Sciences, Boston University, Boston, MA, 02215, USA.

#Yinyi Chang and Jingjing Wang contributed equally to this work.

* Corresponding authors:

Yongmei Song, State Key Laboratory of Molecular Oncology, National Cancer Center/National Clinical Research Center for Cancer/Cancer Hospital, Chinese Academy of Medical Sciences and Peking Union Medical College, Beijing, 100021, China.

Email: symlh2006@163.com; songym@cicams.ac.cn.

Li Sun, National Cancer Center/National Clinical Research Center for Cancer/Cancer Hospital & Shenzhen Hospital, Chinese Academy of Medical Sciences and Peking Union Medical College, Shenzhen, 518116, China.

Email: xjsunli@sina.com.

Zitong Zhao, State Key Laboratory of Molecular Oncology, National Cancer Center/National Clinical Research Center for Cancer/Cancer Hospital, Chinese Academy of Medical Sciences and Peking Union Medical College, Beijing, 100021, China.

Email: zhaozitong880807@126.com.

Dongdong Zhang, State Key Laboratory of Epigenetic Regulation and Intervention, Institute of Biophysics, Chinese Academy of Sciences, Beijing, 100101, China.

Email: dongzhang@ibp.ac.cn.

**This file includes:**

Materials and Methods

Figures. S1-4

Table. S1

## Materials and Methods

### RNA extraction, gDNA extraction, PCR, and qPCR

TRIzol (Invitrogen, USA) was added to the collected cells, frozen tissues, and plasma-derived exosomes, and RNA was extracted using the RNAExpress Total RNA Kit (NCM Biotech, China). The gDNA of the cell lines was extracted using a DNA extraction kit (TIANGEN BIOTECH, China). Reverse transcription was performed using Superscript II Reverse Transcriptase (Invitrogen). RT-qPCR was conducted using BlastaqTM 2X qPCR MasterMix (ABM, Jiangsu, China) using the CFX96 Touch Real-Time PCR Detection System (Bio-Rad, Hercules, California, USA). The internal reference of circRNAs and mRNAs is β-actin, the internal reference of miRNAs is U6, and the external reference of circHIF1α in plasma-derived exosomes is a long-chain noncoding RNA GM13008. The sequences of the qPCR primers used in this study are shown in **Table S1**.

### Colony formation assay

After transfection for 24 h, the cells were inoculated into 6-well plates. After 10 to 14 days, the culture plate was washed with PBS, fixed with methanol for 10 min, and stained with crystal violet dye for 10 min.

### Transwell assay

Migration assay: The transfected cells were resuspended in 100 μL of serum-free

medium and added to the upper chamber. Next, 600 μL of 20% FBS was added to the lower chamber.

Invasion assay: The procedure is similar to the migration assay, but a 2% Matrigel solution should be prepared in serum-free medium in advance and added to the upper chamber.

After incubation, the upper chamber was removed and fixed with methanol for 10 min, followed by incubation with 2% crystal violet solution at room temperature for 10 min. Images were processed and counted using the ImageJ software.

**Drug sensitivity detected by Cell Counting Kit-8 (CCK-8) assay**

After cells were seeded in 96-well plates and then treated with cisplatin at different concentrations for 24h. Cell viability was determined using a CCK-8 assay (NCM Biotech, Suzhou, China). The growth-inhibitory curves were charted by CCK-8, and the half-maximal inhibitory concentration (IC50) representing the cisplatin concentration when cell viability was 50% was calculated.

**Flow cytometry analysis**

*Cell cycle analysis.* After transfection, the cells were treated with overnight starvation for synchronization and then immobile overnight with 70% pre-cooled ethanol. The cells were washed with PBS, stained with propidium iodide (PI, BD Biosciences, NJ, USA), and incubated at room temperature in the dark for 15 min. The proportion of cells in each phase of the cell cycle was determined using flow cytometry (BD Biosciences).

*Cell surface markers analysis.* Cells were digested with 0.05% trypsin, washed with

PBS, and then incubated with straight-labeled antibodies. The cells were then incubated at 4 °C in the dark for 40 min. After centrifugation, the cells were resuspended in PBS. Flow cytometry (BD Biosciences) was used for detection, and FlowJo software was used for data analysis. Flow cytometry was performed using the following antibodies: APC anti-human CD44, APC IgG2b Isotype Control (Proteintech, USA).

**Immunohistochemical (IHC)**

Tumor tissue sections embedded in paraffin were dewaxed with xylene and then hydrated with a gradient concentration of alcohol. The slides were placed in Tris-EDTA buffer (10 mM, pH 8.0), thermally repaired at 100 °C for 15 min, and naturally cooled. Endogenous peroxidase blockers were added and incubated for 20 min. After the sheep serum was sealed for 20 min, it was incubated overnight with primary antibodies (Ki67, CD44, TFRC, SLC40A1, FTH1, SLC3A2, and SLC7A11) at 4 °C. After restoration to room temperature, the samples were incubated with the reaction enhancement solution and secondary antibody for 20 min. DAB color development, hematoxylin counterstaining, dehydration, and sealing were then performed.

**RNA pulldown assays and Mass spectrometry**

Biotin-labeled probes for circHIF1α and control sequences were synthesized *in vitro* (Generay). After rinsing the beads with washing buffer, the sediment was identified using western blot. For mass spectrometry analysis, the precipitate was separated by SDS-PAGE, silver stained with protein stains K (Sango Biotech, China) and sent to Novogene (China) for liquid chromatography-mass spectrometry analysis. The sequences of the probes used are shown in **Table S1**.

**Lentiviral infection**

A luciferase-labeled lentivirus stably knocked down circHIF1α (sh-circHIF1α) and its negative control (sh-NC) were constructed by Genechem (Shanghai, China). The knockdown efficiency of circHIF1α was determined by qPCR.

**Figure legend**

**Figure S1. Identification of circular characteristics and subcellular localization of circHIF1α.**

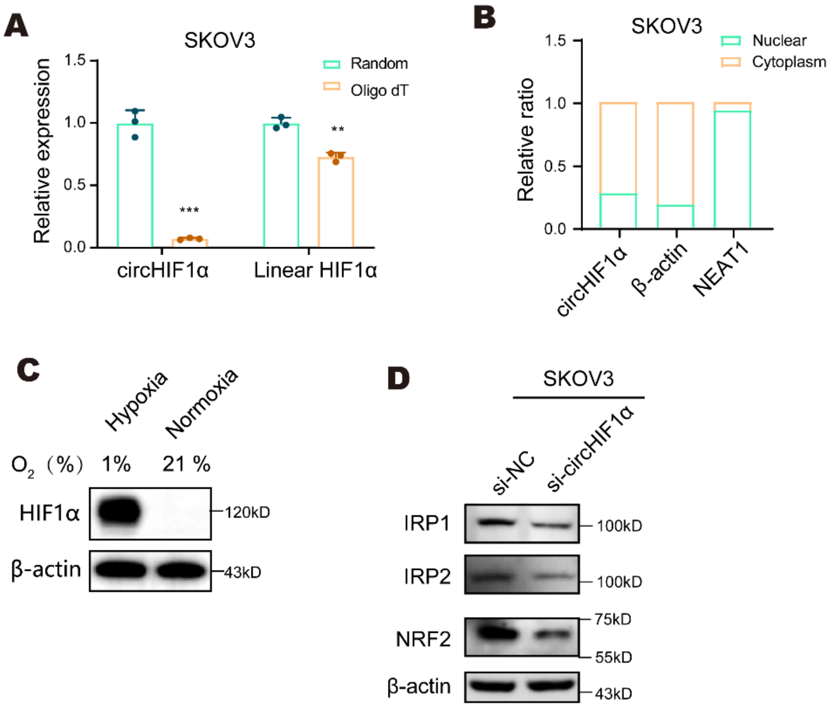


**A**, qPCR analysis of circHIF1α and the linear transcript HIF1α using random primers or oligo-dT primers in SKOV3 cells.

**B**, Subcellular fraction of circHIF1α in the cytoplasm or nucleus of SKOV3 cells. β-actin was used as a cytoplasmic internal reference, and NEAT1 was used as a nuclear internal reference.

**C,** Expression of HIF1α in SKOV3 cells treated with hypoxia or normoxia.

**D**, Western blot analysis of IRP1, IRP2 and NRF2 in SKOV3 cells transfected with si-NC/si- circHIF1α.

The data are presented as the mean ± SD; **, $P < 0.01$; ***, $P < 0.001$.

**Figure S2. CircHIF1α can enhance the stemness of ovarian cancer, promote ferroptosis resistance, chemotherapy resistance and malignant phenotypes *in vitro*.**

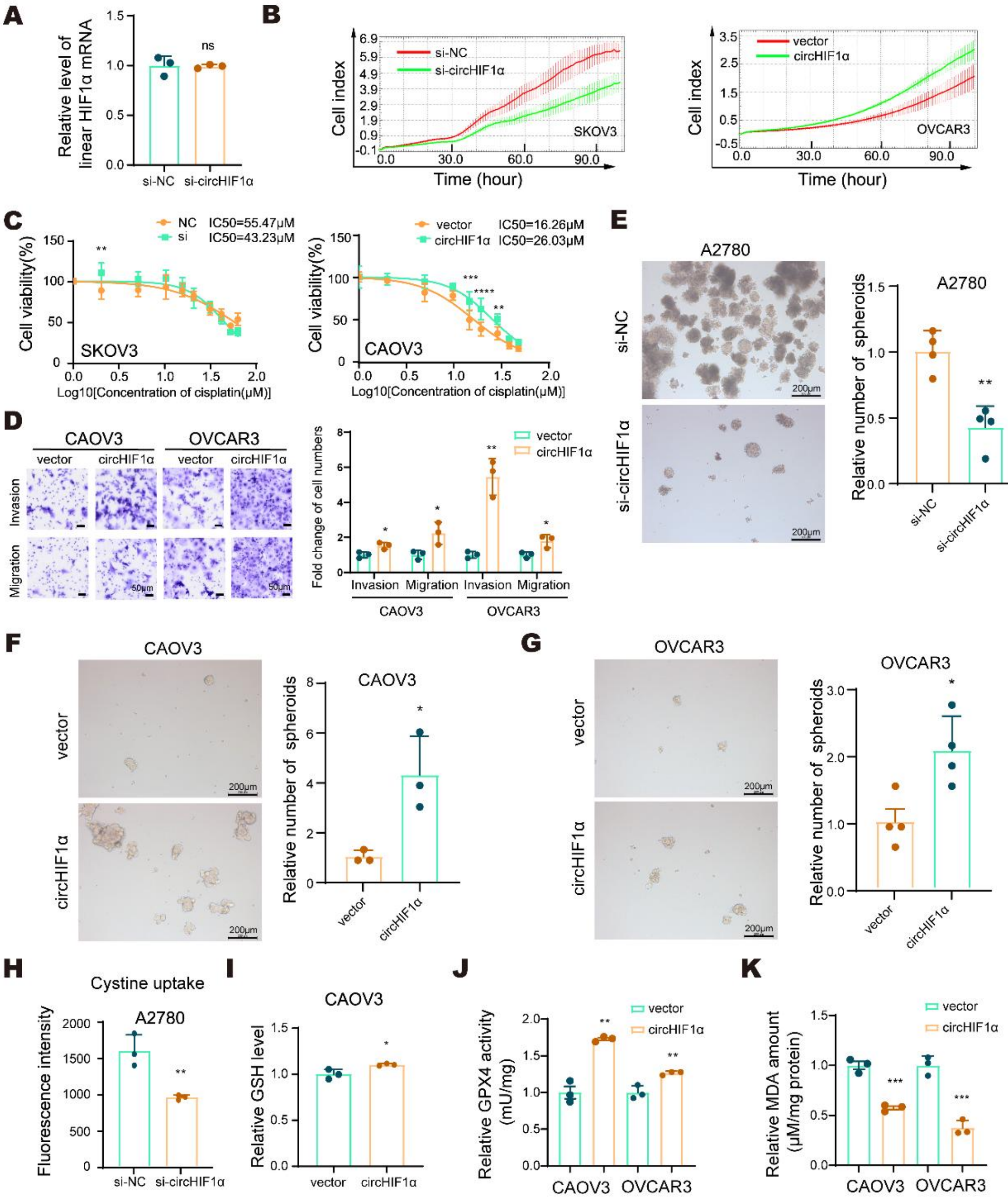


**A**, qPCR analysis of linear HIF1α mRNA in A2780 cells transfected with si-NC/si-circHIF1α.

**B**, Growth of SKOV3 cells transfected with si-NC/si-circHIF1α or OVCAR3 cells transfected with vector/circHIF1α using the xCELLigence Real-Time Cell Analyzer (RTCA)-MP system.

**C**, A CCK-8 assay was performed to evaluate the viability of SKOV3 cells transfected with si-NC/si-circHIF1α or CAOV3 cells transfected with vector/circHIF1α and further treated with various concentrations of cisplatin.

**D**, Transwell assays were performed to analyze the invasion and migration of CAOV3/OVCAR3 transfected with vector/circHIF1α. Representative images of the transwell assay are shown, and quantitative analyses are presented in histograms. Scale bar, 50 μm.

**E-G**, Representative images of spheroids generated from A2780 (**E**), CAOV3 (**F**), and OVCAR3 (**G**) transfected with si-NC/si-circHIF1α or vector/circHIF1α. Scale bar, 200 μm.

**H**, Cystine uptake capacity of A2780 cells transfected with si-NC/si-circHIF1α.

**I**, Relative glutathione (GSH) levels in CAOV3 cells transfected with vector/circHIF1α.

**J**, Relative glutathione peroxidase 4 (GPX4) activity in CAOV3/OVCAR3 cells transfected with vector/circHIF1α.

**K**, Relative malondialdehyde (MDA) amount in CAOV3/OVCAR3 cells transfected with vector/circHIF1α.

The data are presented as the mean ± SD; ns, not significant; *, $P < 0.05$; **, $P < 0.01$; ***, $P < 0.001$; ****, $P < 0.0001$.

**Figure S3. A2780-secreted exosomes harboring circHIF1α enhance stemness maintenance and cisplatin resistance in ovarian cancer.**

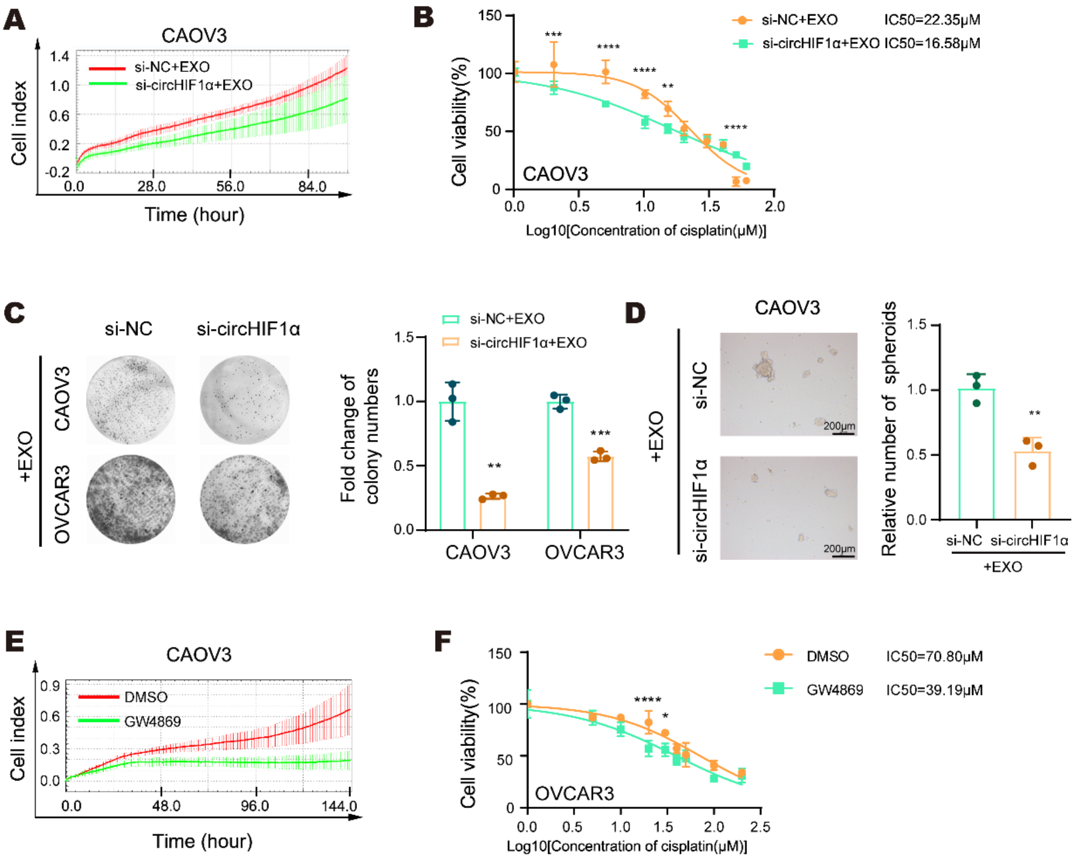


**A**, Growth of CAOV3 cells treated with si-NC/si-circHIF1α and A2780 exosomes using the xCELLigence RTCA-MP system.

**B,** A CCK-8 assay was performed to evaluate the viability of CAOV3 cells transfected with si-NC/si-circHIF1α and A2780 exosomes and further treated with various concentrations of cisplatin.

**C**, Representative images of the colony formation assay are shown and quantitative analyses are presented as histograms.

**D**, Representative images of spheroids generated from CAOV3 transfected with si-NC/si-circHIF1α and A2780 exosomes. The quantitative analyses are presented as histograms on the right. Scale bar, 200 μm.

**E**, Growth of CAOV3 cells treated with A2780-derived culture medium

(DMSO/GW4869).

**F,** A CCK-8 assay was performed to evaluate the viability of OVCAR3 cells treated with A2780-derived culture medium (DMSO/GW4869) and further treated with various concentrations of cisplatin.

The data are presented as the mean ± SD; *, $P < 0.05$; **, $P < 0.01$; ***, $P < 0.001$; ****, $P < 0.0001$.

**Figure S4. Representative images of Hematoxylin-eosin (HE) staining.**

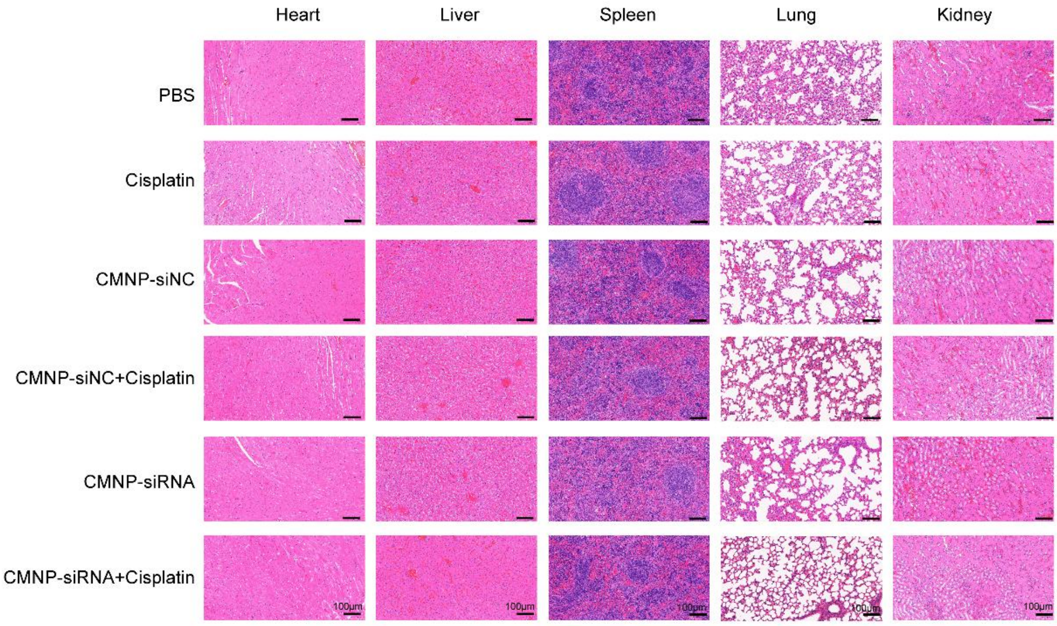


Images of HE staining of the different groups' heart, liver, spleen, lung and kidney. Scale bar, 100 μm.

**Table S1. List of primer and probe sequences.**

| **Gene** | **Sequence (5'-3')** | **Application** |
|---|---|---|
| U6 | F: CTCGCTTCGGCAGCACA<br>R: AACGCTTCACGAATTTGCGT | qPCR |

| | | |
|---|---|---|
| circHIF1α | AGAACTTATCCATTTCTGTG | FISH |
| circHIF1α | ACTTATCCATTTCTGTGTGTA | FISH of tissues |
| circHIF1α | sense probe: AATGCTTACACACAGAAATGGATAAGTTCTGAACGTCGAA antisense probe: TTCGACGTTCAGAACTTATCCATTTCTGTGTGTAAGCATT | RNA pulldown and Mass spectrometry |
| circHIF1α-divergent | F: TCCATGTGACCATGAGGAAA R: TGGCAACTGATGAGCAAGC | qPCR |
| circHIF1α-convergent | F: AGCTTGCTCATCAGTTGCCA R: TCCAAATCACCAGCATCCAGA | qPCR |
| circHIF1α | F: CACAGAAATGGATAAGTTCT R: TGGCAACTGATGAGCAAGC | qPCR |
| β-actin | F: GAAGGTGACAGCAGTCGGTT R: GGACTTCCTGTAACAACGCA | qPCR |
| miR-375 RT | GTCGTATCCAGTGCAGGGTCCGAGGTATTCGCACTGGATACGACTCACGC | qPCR |
| miR-375 | F: CCTTGTTTGTTCGTTCGGCTC R: CGCAGGGTCCGAGGTATTC | qPCR |
| SLC7A11 | F: TCATGGTTGCCCTTTCCCTC | qPCR |

| | | |
|---|---|---|
| | R: TGTTCTGGTTATTTTCTCCGACA | |
| SLC3A2 | F: ACCCCTGTTTTCAGCTACGG<br>R: GGTCTTCACTCTGGCCCTTC | qPCR |
| Gm13008 | F: GCTTTGTGGCCATTGTGCAT<br>R: CATTTGCCCGTCCCAATGTC | qPCR |
| NEAT1 | F: TTTGTGCTTGGAACCTTGCT<br>R: TCAACGCCCCAAGTTATTTC | qPCR |
| VEGFA | F: CACACAGGATGGCTTGAAGA<br>R: AGGGCAGAATCATCACGAAG | qPCR |
| GLUT1 | F: GGCATGGCTTTCCTGTCTCT<br>R: AGCCCAGATACATGGCAGTG | qPCR |
| DDIT4 | F: CATCAGGTTGGCACACAAGT<br>R: CCTGGAGAGCTCGGACTG | qPCR |
| ID1 | F: CTGCTCTACGACATGAACGG<br>R: GAAGGTCCCTGATGTAGTCGAT | qPCR |
| JMJD1A | F: TCAGGTGACTTTCGTTCAGC<br>R: CACCGACGTTACCAAGAAGG | qPCR |
| MCT4 | F: TACATGTAGACGTGGGTCGC<br>R: CTGCAGTTCGAGGTGCTCAT | qPCR |
| IGF2BP3 | F: TCGTGACCAGACACCTGATGAG<br>R: GGTGCTGCTTTACCTGAGTCAG | qPCR |
